# Qualitative Interviews to Better Understand the Patient Experience and Evaluate Patient-Reported Outcomes (PRO) in *RLBP1* Retinitis Pigmentosa (*RLBP1* RP)

**DOI:** 10.1007/s12325-020-01275-4

**Published:** 2020-05-05

**Authors:** Jane Green, Chloe Tolley, Sarah Bentley, Rob Arbuckle, Marie Burstedt, James Whelan, Karen Holopigian, Kali Stasi, Brigitte Sloesen, Claudio Spera, Jean-Yves Deslandes, Anmol Mullins

**Affiliations:** 1 Memorial University of Newfoundland, St. John’s, Canada; 2 Adelphi Values, Bollington, UK; 3 University of Umeå, Umeå, Sweden; 4 Novartis Institute of Biomedical Research, East Hanover, NJ USA; 5 Novartis Institute of Biomedical Research, Cambridge, MA USA; 6 Novartis Pharmaceuticals Corporation, East Hanover, NJ USA; 7 Novartis Pharma AG, Basel, Switzerland

**Keywords:** Interview, Qualitative, Quality of life, *RLBP1* retinitis pigmentosa, *RLBP1* RP, Visual functioning

## Abstract

**Introduction:**

*RLBP1* RP is an autosomal recessive form of retinitis pigmentosa (RP), characterized by night blindness, prolonged dark adaptation, constricted visual fields and impaired macular function. This study aimed to better understand the patient experience of *RLBP1* RP and evaluate the content validity of existing patient reported outcome (PRO) instruments in this condition.

**Methods:**

Semi-structured concept elicitation and cognitive debriefing interviews were conducted with *RLBP1* RP patients in Canada and Sweden. Interviews started with open-ended concept elicitation questioning, and then patients were cognitively debriefed on The National Eye Institute Visual Functioning Questionnaire (NEI VFQ-25), the Low Luminance Questionnaire (LLQ) and four light/dark adaptation items of the Visual Activities Questionnaire (VAQ). Qualitative interviews were also conducted with three expert clinicians. Anonymized, verbatim transcripts were analyzed using thematic analysis.

**Results:**

Twenty-one patients were interviewed (Canada *n* = 10; Sweden *n* = 11). Symptoms reported included night blindness (*n* = 21), difficulty adapting to changes in lighting (*n* = 21) and difficulties seeing in bright lighting (*n* = 18). Patients experienced substantial impacts on daily activities (*n* = 21) and physical functioning (*n* = 17). Patients had difficulty interpreting and selecting a response for some items in the NEI VFQ-25 and LLQ. Some items were not relevant to patients’ disease experience. There were both gaps and overlaps in the conceptual coverage of the instruments.

**Conclusions:**

Visual impairment due to *RLBP1* RP has a substantial impact on physical functioning and daily activities. To adequately assess all important symptoms and associated functional impacts in *RLBP1* RP, it is recommended to either modify one or more existing instruments or to develop a new non-syndromic RP specific instrument.

**Electronic supplementary material:**

The online version of this article (10.1007/s12325-020-01275-4) contains supplementary material, which is available to authorized users.

## Key Summary Points

**Table:** 

**Why carry out this study?**
The symptoms of *RLBP1* RP have a substantial impact on patients’ daily lives and physical functioning.
Semi-structured concept elicitation and cognitive debriefing interviews were conducted with *RLBP1* RP patients (*n* = 21) in Canada and Sweden to better understand the patient experience of *RLBP1* RP and evaluate the content validity of three existing patient reported outcome (PRO) instruments in this condition.
**What was learned from the study?**
Symptoms of *RLBP1* RP reported included night blindness, difficulty adapting to changes in lighting and difficulties seeing in bright lighting, and patients experienced substantial impacts on daily activities and physical functioning.
When asked to complete three different questionnaires, patients with *RLBP1* RP highlighted problems interpreting some questions and response options from all three PRO instruments, and no one instrument provided a comprehensive assessment of concepts relevant to patients with *RLBP1* RP.
To adequately assess all important symptoms and associated functional impacts in this population, either modifying one or more existing PRO instruments or developing a new disease specific PRO instrument is recommended.

## Introduction

*RLBP1* retinitis pigmentosa (*RLBP1* RP) is an autosomal recessive form of retinitis pigmentosa (RP), caused by biallelic mutations of the *RLPB1* gene on chromosome 15. *RLBP1* RP is associated with progressive loss of vision due to retinal degeneration and is a rare disease [1], with only 152–160 cases worldwide reported in the literature [2–18]. Sweden and Canada have the highest number of diagnosed cases, and in each country the cases are clustered in a specific geographic area: Västerbotten County in Sweden and Newfoundland in Canada.

Symptoms of *RLBP1* RP are a result of progressive loss of rod and cone photoreceptor cells [19]. The phenotype caused by mutations identified in *RLBP1* RP is most often referred to as Bothnian dystrophy (BD) [20], Newfoundland rod-cone dystrophy (NFRCD) [5] or retinitis punctata albescens (RPA) [21]. *RLBP1* RP is characterized by severe night blindness from childhood due to prolonged dark adaptation, constricted visual fields (VF) and reduced macular function with decreasing visual acuity (VA) in early adulthood [22]. The impact of *RLBP1* RP on quality of life (QoL) can largely be attributed to visual impairment. Though *RLBP1* RP is limited only to retinal degeneration, it affects patients’ ability to perform vision-dependent functions of everyday life and has a profound effect on physical, social and psychological wellbeing.

A patient-reported outcome (PRO) instrument measures information that comes directly from the patient about the status of their health condition, without amendment or interpretation of the patient’s report by a clinician or anyone else [4, 23]. PRO instruments can assess symptoms, impacts, functioning, and treatment satisfaction and adherence, providing insight into concepts that cannot be measured objectively through biological or functional assessments.

The Food and Drug Administration (FDA) PRO guidance [24] outlines best practices for developing PRO instruments for use to support labeling claims. As a first step in evaluating the appropriateness of an instrument, it is crucial to ensure, through qualitative patient research, that the instrument assesses concepts relevant to the patient population and to ascertain patients’ comprehension of the questionnaire [5, 23].

Although it is understood that *RLBP1* RP affects patients’ visual functioning and therefore activities of daily living (ADL), qualitative evidence of this in the literature is minimal. Currently, no PRO instruments have been validated in an RP population. Several non-disease specific PRO instruments that assess the impact of visual impairment have been used in RP and other visual conditions such as glaucoma, including the National Eye Institute Visual Functioning Questionnaire (NEI VFQ-25) [25, 26], the Low Luminance Questionnaire (LLQ) [27] and the Visual Activities Questionnaire (VAQ) [28].

Previous studies in *RLBP1* RP provide some evidence that NEI VFQ-25 scores are associated with VA, VF and LLQ scores in this population [22, 29, 30]. However, other than these preliminary insights into construct validity, there is limited evidence regarding the content and psychometric validity of these instruments in this specific population. Moreover, neither the NEI VFQ-25 nor the LLQ assesses the process of light-dark adaptation, a key characteristic of *RLBP1* RP. The VAQ contains a four-item dark adaptation subscale.

This study aimed to conduct qualitative research with participants diagnosed with *RLBP1* RP and expert clinicians to: (1) further understand participants’ experiences with visual symptoms and the associated impacts on domains of functioning, (2) evaluate the content validity of the NEI VFQ-25, LLQ and four items from the VAQ (assessing light/dark adaptation) in this population; (3) obtain clinical insight into the experiences of participants with *RLBP1* RP and the clinical relevance of the concepts assessed by the selected instruments.

## Methods

This was a cross-sectional, qualitative study involving individual, semi-structured, concept elicitation (CE) and cognitive debriefing (CD) interviews with patients with *RLBP1* RP. In addition, two pilot interviews were conducted with adult patients who had RP not associated with a mutation in the *RLBP1* gene to test the interview process and allow for comparisons between the two patient groups. Semi-structured interviews were also conducted with expert clinicians with experience of managing and treating patients with *RLBP1* RP.

### Study Sample

Twenty adults and one child (aged 11 years) with *RLBP1* RP were recruited in Canada (*n* = 10) and Sweden (*n* = 11) through expert clinicians specialized in *RLBP1* RP. All participants had a clinical diagnosis of RP with documentation of the *RLBP1* gene mutations. No quotas for representation of subgroups were employed given the rare nature of the condition. The two RP patients participating in the pilot interviews were recruited from Switzerland and Ireland and had been identified via Retina International (an umbrella group of 43 patient-led charities, foundations and voluntary groups funding research into retinal dystrophies). Expert clinicians (*n* = 3) from the recruiting clinical sites in Canada and Sweden were also interviewed.

The adequacy of qualitative sample sizes is commonly evaluated based on the principal of conceptual saturation, defined as the point when no new concept-relevant information emerges with the analysis of additional interviews [31, 32]. Typically, saturation can be achieved with 12 participants; 10 participants per country were targeted because of possible heterogeneity of the populations and to allow for between country comparisons.

### Patient Interview Procedure

The majority of interviews were conducted face-to-face; however, the two participants in the pilot interviews and two participants in Canada were interviewed via Skype. The patient interviews lasted approximately 90-min (45-min concept elicitation and 45-min cognitive debriefing) using a semi-structured interview guide. Concept elicitation was completed prior to review of the PRO instruments to avoid bias.

Concept elicitation questioning explored participants’ experiences of *RLBP1* RP. Questioning started with broad open-ended questions designed to elicit spontaneous comments, followed by focused questions to probe on topics of interest.

The NEI VFQ-25, LLQ and four items from the VAQ were then cognitively debriefed. These instruments were selected from a review of existing PRO instruments that have previously been used in RP and similar conditions. The review considered prior use of the instruments and evidence of content and psychometric validity. The instruments selected had the greatest potential to assess the concepts most important and relevant to RP patients. Cognitive debriefing involved use of a ‘think aloud’ approach, where participants spoke their thoughts aloud as they completed each item. Participants were also asked debriefing questions to explore their interpretation of the items and response options, to evaluate the relevance of concepts assessed by the instruments and to assess conceptual comprehensiveness of the instruments.

### Clinician Interview Procedure

The 30-min clinician interviews were conducted via telephone. One was conducted prior to patient interviews to help inform the content of the patient interview guide, and two were conducted following patient interviews to assess the clinical relevance of reported concepts. Key symptoms and functional impacts of *RLBP1* RP were discussed, as were clinicians’ perceptions of the appropriateness of the NEI VFQ-25, LLQ and four items from the VAQ for use in the *RLBP1* RP population.

### Analysis

Interviews were anonymized and transcribed verbatim. Swedish transcripts were translated to English. Qualitative analysis was performed using Atlas.Ti software [33] and thematic analysis methods [34–36]. For concept elicitation, this focused on identifying concepts important to participants and the language participants used to describe those concepts. Saturation (the point at which no new concepts are emerging from the data) was evaluated by comparing spontaneously reported concepts from successive sets of interviews. A conceptual model was generated to summarize the key concepts relevant for assessment in *RLBP1* RP. A conceptual model is useful for presenting a concise overview of the patient experience of a condition and for prioritizing, identifying and evaluating measurement strategies [37].

### Ethical Considerations

Ethical approval was obtained from the Newfoundland and Labrador Health Research Ethics Board in Canada (reference no. 2016.224) and Umea University in Sweden (reference no. 2016/357-31). Written informed consent from each participant was obtained prior to data collection.

## Results

### Patient Sample Characteristics

Demographic and clinical characteristics of the *RLBP1* RP sample are summarized in Table 1. Mean age was 45 years (range 11–67 years), and there were marginally more males (male 12/21, female 9/21). According to clinical data provided by clinicians, participants were diagnosed with *RLBP1* RP on average 14 years after experiencing initial symptoms of RP. Participants were assigned severity ratings (mild, moderate, severe or very severe) based on their VA score and Visual Field Index (VFI). The VA scores were converted to a severity rating based on the adjusted World Health Organization (WHO) categories of visual impairment [38]. The VFI categories were informed by published data regarding VFI severity categories in glaucoma [39]. Visual function scores for right and left eyes were summarized into a single weighted average where the better eye is weighted 0.75 and the worse eye 0.25. The sample included a wide range of VA severities, but most had severe or very severe VFI ratings (Table 2).

**Table 1:** Patient demographic and clinical information

Characteristic	Canada (*n* = 10)	Sweden (*n* = 11)	Total (*n* = 21)
Gender, *n*			
Male	5	7	12
Female	5	4	9
Age (years)			
Mean	46.7	43.7	45
Range	29–65	11–67	11–67
Race, *n*			
Caucasian	10	11	21
Living status, *n*			
Lives alone	1	0	1
Lives with husband/wife/partner	8	6	14
Lives with children	3	2	5
Lives with parents/other family members (note: more than one option can be selected)	1	2	3
Work status, *n*			
Working full time	5	3	8
Working part time	0	1	1
Full time homemaker	0	2	2
Student	0	2	2
Not working due to *RLBP1* RP	4	1	5
Retired (note: one participant was a child)	1	1	2
Years between initial *RLBP1* RP symptoms and diagnosis			
Mean (range)	20.9 years (2–62)	7.7 years (1–27)	14 years (1–62)
Median	18 years	5 years	13 years
Weighted visual acuity^a^			
Mean (range)	15.6 letters (0–54)	35.6 letters (1–88)	26 letters (0–88)
Weighted visual field index^a^			
Mean (range)	17.9% (22–79)	38.3% (0–97)	29.6% (0–97)

^a^Visual function scores for left and right eyes were summarized into a single weighted average where the better eye is weighted 0.75 and the worse eye 0.25

**Table 2:** Severity ratings

Visual field	Visual acuity			
	Mild	Moderate	Severe	Very severe
Mild	1			
Moderate		1		
Severe	3		2	
Very severe		2	4	8

### Patient Concept Elicitation Results

**Fig. 1:**
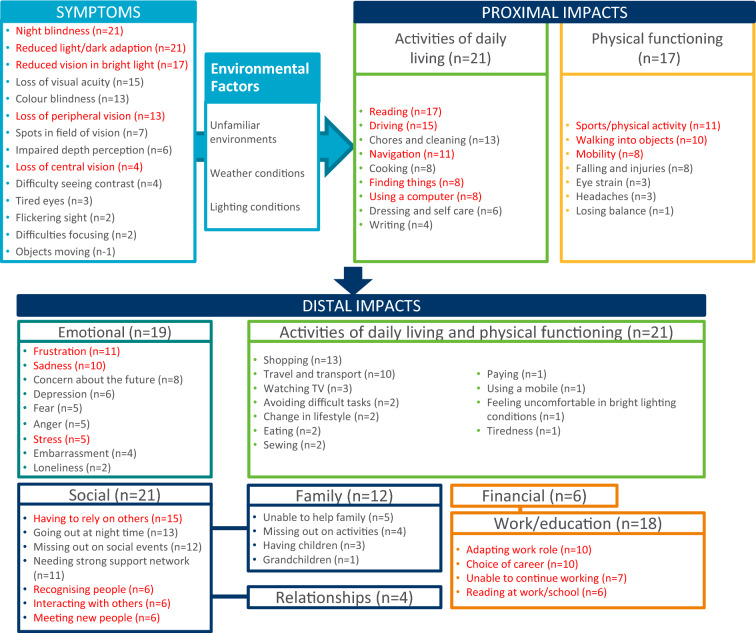
Conceptual model for *RLBP1* RP. Concepts in red were considered ‘key concepts’ by expert clinicians

Figure 1 presents the conceptual model developed based on the interview findings to illustrate the key concepts and impact on functioning relevant to patients with *RLBP1* RP. Fourteen visual symptoms were reported during concept elicitation. The symptoms most frequently discussed were:

- Night blindness (*n* = 21; all spontaneously reported). The majority of participants described being unable to see anything in dark conditions (*n* = 15/21). The remaining six participants reported that they had limited vision in darkness. Those that described being unable to see at all used phrases such as “can’t see anything” and “can’t see at all” (*n* = 10/15), “night blindness” (*n* = 4/15) and “see absolutely nothing” (*n* = 1/15).
  “For me when it gets darker, I see less and less. And all of a sudden, I can’t see anything at all” (Male, 20).
- Difficulties adapting to changes in lighting (*n* = 21; all spontaneously reported). All of the participants reported that they had difficulties adapting to changes in light when moving from brightly lit areas to dimly lit areas, and/or vice versa. Eleven participants reported difficulty adapting in either direction; eight participants described difficulty moving from light to dark areas only, and two participants described difficulty moving from dark to light areas only.
  “Sometimes if I leave a dark room and enter say a lit office, that can be sometimes a little bit excruciating” (Male, 40).
- Difficulties seeing in bright lighting (*n* = 18; spontaneous *n* = 15, probed *n* = 3). Participants reported that in bright lighting conditions they were no longer able to see (*n* = 12/18) or found it more difficult to see (*n* = 2/18). Five participants also reported that they were ‘sensitive’ to bright light (*n* = 5/18). Terminology used included ‘blinded’ (*n* = 8/18), ‘can’t see’ or ‘difficulty seeing’ (*n* = 3/18). Five participants also referred to this symptom as difficulty with ‘glare.’
  “Sunlight is the worst, it’s the most intensive. It blinds me the most” (Male, 25).
- Poor visual acuity (*n* = 15; all spontaneously reported). Participants used a variety of terms to describe low visual acuity. Approximately half of participants (*n* = 8/15) directly used the term “low visual acuity.” Other terms used included describing their vision becoming ‘blurry’ (*n* = 2/15), ‘farsighted’ (*n* = 1/15) and ‘myopic, near-sighted’ (*n* = 1/15).
  “I have sight loss, my visual acuity has decreased. Especially in the middle of the eye, it’s becoming increasingly difficult to read” (Female, 28).
- Impaired color vision (*n* = 13; spontaneous *n* = 11, probed *n* = 2). Thirteen participants described difficulty in seeing and distinguishing between colors. Six participants discussed the specific colors that they have a problem distinguishing. The majority (*n* = 5/6) reported that they are unable to distinguish between dark colors, e.g., between a navy blue and black.
  “I’ve lost the part you see colors with, so I don’t see colors. I see TV in black and white” (Female, 58).
- Loss of peripheral vision (*n* = 13; spontaneous *n* = 9, probed *n* = 4). Thirteen participants reported experiencing difficulties with their peripheral vision, eight of whom used or were familiar with the term ‘peripheral vision’ (*n* = 8/13). Four participants (*n* = 4/13) described instances where they were unable to see objects when they were presented to the side of their visual field. Two participants (*n* = 2/13) additionally used the terms ‘tunneling’ (*n* = 1/13) or ‘tunnel vision’ (*n* = 1/13) to describe their visual field.
  “It seems like just tunnel vision is all I can see. I can’t really see in the side of me much anymore, it’s just kind of straight, central” (Female, 35).

Participants across the sample appeared to experience a similar core set of symptoms of *RLBP1* RP that worsened in severity over time. Participants also described environmental factors that affected the symptoms reported; these included weather and lighting conditions (*n* = 16/21) and unfamiliar environments (*n* = 16/21).

All participants discussed the impact of *RLBP1* RP on ADL. Impacts were separated into proximal impacts and distal impacts. Proximal impacts were considered to be any functional impacts directly related to vision impairments associated with *RLBP1* RP (e.g., reading). Distal impacts were considered to be any impacts that occurred as a result of the proximal impacts that had wider implications for participants’ daily life (e.g., work, reading menus, etc.).

The most frequently reported proximal impacts on ADL included:

- Difficulty reading (*n* = 17; spontaneous *n* = 12, probed *n* = 5). Impact on ability to read was affected by font style (*n* = 6/17), size of the text (*n* = 4/17) and the color contrast of the text and background (*n* = 3/17). Reduced reading ability had a subsequent impact on various aspects of life such as work, education, social life, shopping and cooking. Three participants also expressed difficulty in navigating because of their reduced ability to read signs. Methods to manage this impact on reading were discussed by seven participants and included using electronic devices such as computers, use of magnifiers and use of audio books.
  “I read a lot using a tablet where you can zoom in the text” (Female, 28).
- Being unable to drive or having difficulties driving (*n* = 15; all spontaneously reported, only one participant was still able to drive). The remaining six participants did not discuss the impact of *RLBP1* RP on their ability to drive and were not asked. Reasons for being unable to drive described by 14 participants included poor nighttime vision (*n* = 5/14), the eyes not being able to adjust (*n* = 1/14), experiencing blind spots (*n* = 1/14) and defects in peripheral vision (*n* = 2/14). Four participants reported the impact of not being able to drive on their daily life; these included the need for advanced planning and the impact on family life. Five participants talked about requiring assistance from family and friends to drive them around. Four participants reported feelings of frustration, sadness, depression and devastation due to their inability to drive.
  “Something that is very bothersome is that I don’t have a driver’s license. I can’t drive” (Male, 54).
- Problems with chores and cleaning (*n* = 13; spontaneous *n* = 12, probed *n* = 1): Participants expressed the need for assistance in carrying out chores and cleaning (*n* = 6/13). Participants also made clear that their ability has deteriorated overtime (*n* = 3/13), with chores taking considerably longer to complete (*n* = 3/13). Participants also mentioned the importance of adaptations and routines when completing chores (*n* = 4/13).
  “Cleaning too is another one. I miss a lot of things because I can’t see a lot of the things” (Female, 35).
- Problems navigating in unfamiliar environments (*n* = 11; all spontaneously reported). Participants indicated that difficulty to navigate was often caused by darkness (*n* = 5/11) and the inability to see obstacles (*n* = 5/11). Five participants talked about specific strategies they often use when navigating. Such strategies included following familiar streetlights or house lights, use of technology, being organized and memorizing routes. Difficulties navigating affected participants ability to travel to appointments (*n* = 1/11) and their social life (*n* = 1/11).
  “At the house it’s not too bad because I know where everything is, but especially if I were in a strange place, I’d need some form of lighting or know the layout of the room. Otherwise, I could fall down or trip over something” (Male, 47).

The most frequently reported proximal physical impacts included:

- Reduced ability to take part in sports (*n* = 11; spontaneous *n* = 3, probed *n* = 8). Eight of the 11 participants reported that they were unable to take part in certain sports and activities, and three participants reported that they could still take part but were limited in their ability. Examples of sports and activities reported by more than one participant included: hockey (*n* = 5/11), football (*n* = 3/11), riding a bike (*n* = 3/11), baseball (*n* = 2/11) and swimming (*n* = 2/11).
  “I do some sport, if it’s adapted, but, you know, regular sports are not really an option” (Male, 40).
- Walking into objects (*n* = 10; all spontaneously reported): More frequently reported examples of objects that participants had walked into because of their impaired vision included: obstacles associated with road works (*n* = 3/10), other people (*n* = 3/10) and objects on the floor at home (e.g., furniture and bins; *n* = 2/10).
  “We would do things together, and I could not see on this side, and we would bump into each other all the time” (Female, 58).
- Having difficulties with their mobility due to poor vision (*n* = 8; all spontaneously reported): Three of the eight participants reported that they had difficulty generally getting around going to work or the supermarket, three participants reported difficulties going up or down stairs, one participant reported difficulties with outdoor activities, and one participant reported having to ‘walk more carefully.’
  “I guess steps, the depth, the difference between the steps… I can’t see going down steps, but I can see sometimes going up steps” (Female, 63).

Wider impacts on quality of life were also identified, including:

- Impacts on social functioning (*n* = 21; all spontaneously reported). Social impacts included having to rely on others for help (*n* = 14/21), difficulties going out at night time to social events (*n* = 13/21), missing out on social events (*n* = 12/21), problems recognizing people (*n* = 6/21), problems attending the cinema (*n* = 6/21) or restaurants (*n* = 5/21), being unable to help family members (*n* = 5/21), missing out on activities with children/grandchildren (*n* = 4/21) and impacts on current/future relationships (*n* = 4/21).
  “Uh, well, uh, I guess, I used to, uh, go out with friends and to parties and such and I usually depended on some of them to, uh, guide me around” (Male, 29).
- Emotional wellbeing (*n* = 19; all spontaneously reported). Emotions experienced by participants because of their condition in order of frequency included frustration (*n* = 11/19), sadness (*n* = 10/19), concerns about the future (*n* = 8/19), depression (*n* = 8/21), fear (*n* = 5/19), anger (*n* = 5/19), stress (*n* = 5/19), embarrassment (*n* = 4/19) and loneliness (*n* = 2/19).
  “So, I just feel like I’m a big burden on people a lot of the times, and it’s very frustrating. It gets very emotional for me” (Female, 35).
- Work and education (*n* = 18; spontaneous *n* = 13, probed *n* = 5). Participants described adapting their work role because of their condition (*n* = 10/18), having a limited choice of career or job (*n* = 10/18), being unable to continue working in a particular job (*n* = 7/18) and having difficulties at work related to their ability to read (*n* = 6/18).
  “And then my workday, like I said, I’ve switched my duties so that I can be, um, able to do my job without having need perfect vision. So, I’m in my office most of the day. I do have, um, a setting on my computer that reverses the contrast of the lighting so it’s not so bright” (Female, 32).

An important aspect of the qualitative analysis was to evaluate the degree of consistency in the findings to determine whether conceptual saturation had been achieved among the concept elicitation interviews and that all concepts of importance had been identified. It was concluded that saturation was achieved for all core symptoms and impacts of *RLBP1* RP.

As would be expected, participants with more severe *RLBP1* RP (with regards to visual acuity and visual fields) appeared to have a more severe experience of certain symptoms. For example, participants categorized as severe and very severe took longer to adapt to changes in lighting than mild or moderate participants. In general, participants categorized as severe and very severe reported a greater impact of *RLBP1* RP compared with mild and moderate participants. Minor differences in impacts experienced by the Swedish and Canadian populations were observed. These are likely a result of differences in the sample severities or lifestyles of the two populations rather than differences in disease manifestation.

### Pilot Interview Findings

The findings from the interviews with RP patients did not identify any notable differences between the experiences of patients with *RLBP1* RP and those with ‘general RP’ interviewed in pilot interviews. However, as only two pilot interviews were conducted, conclusions regarding these comparisons should be interpreted with caution.

### Cognitive Debriefing Results

Challenges interpreting items identified for the NEI VFQ-25 and LLQ are summarized in Table 3. Detailed cognitive debriefing results for the instruments are provided as supplementary files (NEI VFQ-25: Supplementary file 1; LLQ: Supplementary file 2; VAQ: Supplementary file 3).

**Table 3:** Summary of key difficulties related to interpretation of the NEI VFQ-25 and the LLQ

Summary of issue	Number of items flagged as problematic in the NEI VFQ-25 and example	Number of items flagged as problematic in the LLQ and example
No specification of lighting conditions or familiarity of the environment	7/25 items were flagged Example: Because of your eyesight, how much difficulty do you have finding something on a crowded shelf? (Item 7) 9/21 participants reported that they would have difficulty selecting a response as their ability is affected by the lighting conditions	3/32 items were flagged Example: Do you have difficulty seeing when you visit other people’s homes because there is not enough light? (Item 19) 7/21 participants had difficulty interpreting the item stating that their visual ability is dependent on how familiar the environment is and lighting conditions in those environments
Some items provided multiple examples of activities which represented different levels of functional impairment	4/25 items were flagged Example: Because of your eyesight, how much difficulty do you have visiting with people in their homes, at parties or in restaurants? (Item 13) 8/21 participants reported that the examples included in the item would result in different responses	3/32 items were flagged Do you have difficulty seeing in poor lighting conditions such as at dusk or dawn or in a poorly lit room? (Item 16) 5/21 participants reported that there were differences in their visual ability in dusk, dawn and poorly lit rooms, making it difficult for them to decide how to respond
Response options stating ‘stopped doing this’ for specific activities were not always appropriate	8/25 items were flagged Example: Because of your eyesight, how much difficulty do you have noticing objects off to the side while you are walking along? (Item 10) 6/21 participants reported that they would never stop trying to notice objects off to the side	12/32 items were flagged Example: Do you have difficulty seeing colors at night? (Item 13) 4/21 participants reported that they would not stop trying to see color
Examples provided in the item were outdated	1/25 items were flagged Example: How much difficulty do you have doing work or hobbies that require you to see well up close, such as cooking, sewing, fixing things around the house or using hand tools? (Item 6) 3/21 participants suggested that more modern examples of tasks which require the ability to see up close should be used	1/32 items were flagged Example: Because of your vision, do you have difficulty going out to nighttime social events such as sporting events, the theater, friend’s homes, church or restaurants? (Item 10) 1/21 suggested that the examples should be modernized

### Interpretation of the NEI VFQ-25

For 19 of the 25 items of the NEI VFQ-25, at least some of the participants had difficulty interpreting the item or choosing a response, most often because of a lack of specification of the lighting conditions or whether the environment was familiar (Table 3). Additionally, some items included multiple examples of activities that represented different levels of functional impairment, making it difficult to choose a response. Finally, some response options were inappropriate (e.g., a response option of ‘stopped doing this’ for items assessing difficulty noticing objects off to the side’), and some items described examples of tasks that were outdated. Note that items related to driving were not debriefed with all participants given the skip patterns built into the NEI VFQ-25.

Feedback for item 13 “Because of your eyesight, how much difficulty do you have visiting with people in their homes, at parties or in restaurants?”:

“Restaurants I find bad… But in homes, friends, like I said, they know I’m coming, they’ve got an extra few lights on or things like that, and a strange home, yes, I’d, I’d have a lot of trouble… If it’s in my friends’ house, a moderate difficulty, but someone else’s would be extreme difficulty” (Male, 50).

### Relevance of the NEI VFQ-25

In total, 22/25 of the NEI VFQ-25 items were relevant to over half of the 21 participants. Items were classed as relevant if a participant had ever experienced the impairment or functional limitation, even if they did not currently experience it. The items that were least relevant were item 4 (*n* = 16) and item 19 (*n* = 17), which both assess pain and discomfort around the eyes. Item 15, “Are you currently driving at least once in a while?,” was only relevant to one participant, as only one participant could still drive.

Feedback for item 7 “Because of your eyesight, how much difficulty do you have finding something on a crowded shelf?”:

“If the question had mentioned light, if it’s difficult to find things on a crowded shelf when you come from outside. Or in a shop with poor lighting, is it difficult to find things, that would have been relevant” (Male, 25).

### Interpretation of the LLQ

For 24 of 32 items of the LLQ, at least some of the participants had difficulty interpreting the item or choosing a response. Similar to the NEI VFQ-25, participants had issues interpreting and choosing a response to items because lighting conditions and familiarity of environment were not specified or because the examples of activities listed in the questions correspond to different levels of functional impairment (Table 3).

Feedback for item 19 “Do you have difficulty seeing when you visit other people’s homes because there is not enough light?”:

“But where I go most people know and the lights are on, so, you know, I would say a lot of difficulty based on if I were to go somewhere and it was dimly light, somebody else’s house, unfamiliar surroundings it would be a lot of, a lot of difficulty” (Female, 32).

### Relevance of the LLQ

All of the LLQ items were relevant for over half of the 21 participants, with the exception of seven items related to driving. Apart from the driving items, the two least relevant items were item 2, “Do you have difficulty seeing in fluorescent lighting, like that found in stores and offices?,” and item 12, “Do you worry or are you concerned that you might fall at night because of your vision?” Of note, several participants reported that they were more likely to fall but were just not concerned or worried about it; hence, item 12 was considered less relevant:

“That’s not saying that I might not fall, it’s just that I’m not worried about it” (Male, 47).

### Interpretation and Relevance of the Four VAQ Items

Participants had no difficulty interpreting the four VAQ items assessing light/dark adaptation. The two items assessing adaptation from bright to dim/dark-light (item 12 “It takes me a long time to adjust to darkness after being in bright light” and item 28 “I have trouble adjusting from bright to dim lighting, such as when going from daylight into a dark movie theater”) were relevant to all 21 participants. The two items assessing adaptation from dim to bright light (item 1 “I have problems adjusting to bright room lighting, after the room lighting has been rather dim” and item 23 “It takes me a long time to adjust to bright sunshine after I have been inside a building for a lengthy period of time”) were relevant for 17 of the 21 participants.

### Pilot Interview Findings

The findings from the two pilot interviews with RP patients did not identify any notable differences between the experiences of patients with *RLBP1* RP and those with ‘general RP.’ Furthermore, there were no substantial differences in item interpretation and relevance of concepts between participants with *RLBP1* RP and RP. However, as only two pilot interviews were conducted, conclusions regarding these comparisons should be interpreted with caution.

### Conceptual Coverage

Table 4 summarizes the extent to which each of the instruments being evaluated assesses the concepts that were identified as important through concept elicitation interviewing. No instrument alone assesses all concepts relevant to *RLBP1* RP patients. Notably, the NEI VFQ-25 and LLQ do not assess dark/light adaptation, a key defining symptom of *RLBP1* RP; the NEI VFQ-25 does not assess vision in bright light or depth perception, which are important concepts in *RLBP1* RP. There are also gaps in the conceptual coverage of all three instruments in relation to functional impacts, particularly distal impacts on ADLs and impacts on physical functioning.

**Table 4:**
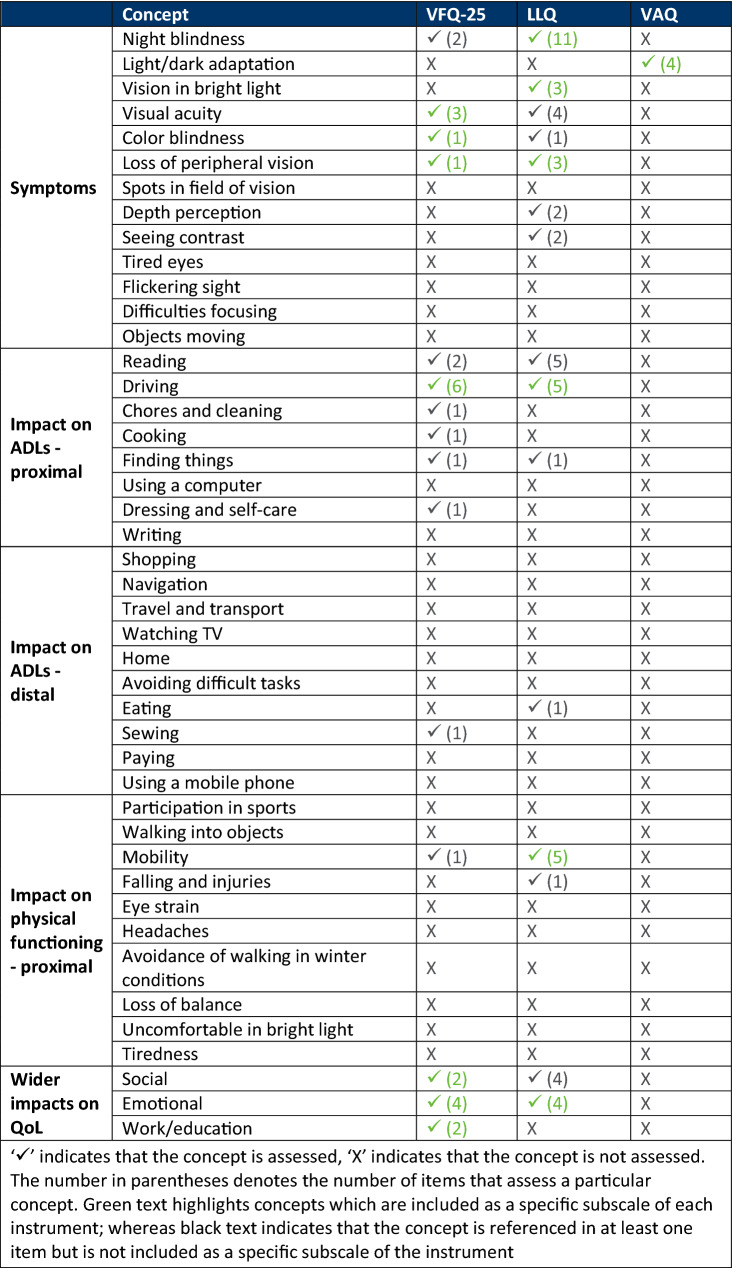
Summary of conceptual coverage of instruments

### Expert Clinician Interviews

All clinicians reported that severity of symptoms varies among *RLBP1* RP patients, with the condition generally becoming more severe with age. The symptoms and impacts reported by the expert clinicians were broadly consistent with those reported by the patients. The experts also highlighted the loss of independence experienced by patients and confirmed the importance of environmental factors such as familiarity of environment and lighting conditions in mediating the impact of patients’ visual impairment. The expert clinicians commented that the symptoms of *RLBP1* RP are widely similar to those of broader RP, but that *RLBP1* RP patients start to lose central vision at an earlier age. The experts identified issues with the NEI VFQ-25 and LLQ that were consistent with those reported by patient participants.

## Discussion

To our knowledge, this is the first comprehensive qualitative study of the patient experience of *RLBP1* RP. The symptoms and impacts reported in this study were consistent with those reported in the scientific literature relating to RP and *RLBP1* RP and consistent with reports from expert clinicians [5, 19, 20, 22, 40, 41]. However, this study provides a more in-depth understanding of the patient experience of *RLBP1* RP from the perspective of patients themselves than was previously available. Based on the literature, two RP patient pilot interviews and clinician interviews, it seems likely that the findings reported here may be generalized to RP as well as *RLBP1* RP [42, 43]. However, further study in a sample of RP patients without the *RLBP1* gene mutations is necessary to confirm whether the findings from this study can be extrapolated to the broader population.

Neither the NEI VFQ-25, the LLQ, nor the four items tested from the VAQ provide a comprehensive assessment of the *RLBP1* RP patient experience, even if administered in combination. In addition, the inconsistencies in item and response option interpretation and limited relevance of some items suggest that, at a minimum, some revision of those instruments is recommended for use in this population.

While some previous studies have demonstrated an association between visual function scores and scores on the instruments in this population, thus supporting construct validity [22, 29, 30], other studies have highlighted issues with the face validity and psychometric properties of the instruments in other similar populations [44–48]. For example, a previous study in patients with Birdshot chorioretinopathy highlighted that the composite score of the NEI VFQ-25 was not statistically associated with decreased visual acuity. Results also suggested that the NEI VFQ-25 may have poor sensitivity at the lower end of the severity scale and may be prone to placebo effects. In addition, previous studies suggest, consistent with findings here, that the LLQ would not provide a full picture of the vision related QoL limitations of *RLBP1* RP patients.

Since this research was conducted, the multi-luminance mobility test (MLMT), a performance related outcome assessment, has been developed and validated to assess ADLs in retinal diseases; data from performance outcome assessments such as the MLMT are valuable to complement data generated via PRO instruments [49, 50].

The findings reported provide valuable insight into the patient experience of *RLBP1* RP and the assessment of content validity in three visual function PRO measures. However, findings should be interpreted in light of the limitations of the study. The sample size of the study is relatively small for qualitative research; however, the sample size reflects the rare nature of *RLBP1* RP. Furthermore, based on the literature and two pilot interviews, it seems likely that the findings reported may generalize to RP as well as *RLBP1* RP. However, for that claim to be credible it would be important to replicate the study in a larger sample of RP patients with a variety of mutations. Additional research doing just this is currently on-going. The research was conducted with patients recruited from Canada and Sweden in centers with a high prevalence of *RLBP1* RP. Further research to confirm the generalizability of the findings for other cultures is warranted, particularly in relation to functional impairment, which may vary by country. Finally, the participants involved in the study were considered to have severe or very severe visual function limitations consistent with the early onset and rapid progression of *RLBP1* RP; therefore, results may not fully represent younger participants with mild or moderate visual function limitations in *RLBP1* RP. However, representation from each visual function severity level was obtained, and differences associated with the severity level of *RLBP1* RP were consistent with those described by clinicians.

## Conclusion

This study has identified issues with conceptual coverage, relevance and participant interpretability of items in the NEI VFQ-25, LLQ and VAQ in the context of *RLBP1* RP. This is consistent with evidence from other studies regarding the inadequacy of these instruments in RP and similar diseases [51].

These findings suggest that modification of one or more existing instruments or development of a new non-syndromic RP specific instrument is warranted to adequately assess the symptoms and associated impacts in *RLBP1* RP. Such an instrument would allow generation of data that would be of value to regulators, payers, prescribing clinicians and, most importantly, patients.

## Electronic Supplementary Material

Below is the link to the electronic supplementary material.

Supplementary material 1 (DOCX 662 kb)

Supplementary material 2 (DOCX 508 kb)

Supplementary material 3 (DOCX 405 kb)

## References

[1] Council NC (2004). NICE Citizens Council Report: ultra orphan drugs.

[2] Botelho PJ, Blinder KJ, Shahinfar S (1999). Familial occurrence of retinitis punctata albescens and congenital sensorineural deafness. Am J Ophthalmol.

[3] Demirci FYK, Rigatti BW, Mah TS, Gorin MB (2004). A novel compound heterozygous mutation in the cellular retinaldehyde-binding protein gene (RLBP1) in a patient with retinitis punctata albescens. Am J Ophthalmol.

[4] Dessalces E, Bocquet B, Bourien J, Zanlonghi X, Verdet R, Meunier I (2013). Early-onset foveal involvement in retinitis punctata albescens with mutations in RLBP1. JAMA Ophthalmol.

[5] Eichers ER, Green JS, Stockton DW, Jackman CS, Whelan J, McNamara JA (2002). Newfoundland rod-cone dystrophy, an early-onset retinal dystrophy, is caused by splice-junction mutations in RLBP1. Am J Hum Genet.

[6] Fishman GA, Roberts MF, Derlacki DJ, Grimsby JL, Yamamoto H, Sharon D (2004). Novel mutations in the cellular retinaldehyde-binding protein gene (RLBP1) associated with retinitis punctata albescens. Arch Ophthalmol.

[7] Flynn M, Bohnert D (1999). Fundus albipunctatus and other flecked retina syndromes. J Am Optom Assoc.

[8] Genead MA, Fishman GA, Lindeman M (2010). Spectral-domain optical coherence tomography and fundus autofluorescence characteristics in patients with fundus albipunctatus and retinitis punctata albescens. Ophthalmic Genet.

[9] Hipp S, Zobor G, Glöckle N, Mohr J, Kohl S, Zrenner E (2015). Phenotype variations of retinal dystrophies caused by mutations in the RLBP1 gene. Acta Ophthalmol.

[10] Zhang X, Wen F, Hu S, Yan H, Lin S (2004). Fundus angiography findings of atypical retinitis pigmentosa. Chin Ophthalmic Res.

[11] Humbert G, Delettre C, Sénéchal A, Bazalgette C, Barakat A, Bazalgette C (2006). Homozygous deletion related to Alu repeats in RLBP1 causes retinitis punctata albescens. Investig Ophthalmol Vis Sci.

[12] Katsanis N, Shroyer N, Lewis R, Cavender J, Al-Rajhi A, Jabak M (2001). Fundus albipunctatus and retinitis punctata albescens in a pedigree with an R150Q mutation in RLBP1. Clin Genet.

[13] Maw MA, Kennedy B, Knight A, Bridges R, Roth KE, Mani E (1997). Mutation of the gene encoding cellular retinaldehyde–binding protein in autosomal recessive retinitis pigmentosa. Nat Genet.

[14] Morimura H, Berson EL, Dryja TP (1999). Recessive mutations in the RLBP1 gene encoding cellular retinaldehyde-binding protein in a form of retinitis punctata albescens. Investig Ophthalmol Vis Sci.

[15] Nakamura M, Lin J, Ito Y, Miyake Y (2005). Novel mutation in RLBP1 gene in a Japanese patient with retinitis punctata albescens. Am J Ophthalmol.

[16] Naz S, Ali S, Riazuddin SA, Farooq T, Butt NH, Zafar AU, Khan SN, Husnain T, MacDonald IM, Sieving PA, Hejtmancik JF (2011). Mutations in RLBP1 associated with fundus albipunctatus in consanguineous Pakistani families. Br J Ophthalmol.

[17] Nojima K, Hosono K, Zhao Y, Toshiba T, Hikoya A, Asai T (2012). Clinical features of a Japanese case with Bothnia dystrophy. Ophthalmic Genet.

[18] van Genderen MM, Littink KW, van Schooneveld, MJ, Riemslag FCC, Keunen JEE, den Hollander AI, et al. Genotypes and phentotypes in fundus albipunctatus and retinitis punctata albescens [abstract]. In: Proceedings of the 50th ISCEV International Symposium; 5–8 June 2012; Valencia, Spain. Abstract number OS2-4.

[19] Hartong DT, Berson EL, Dryja TP (2006). Retinitis pigmentosa. Lancet.

[20] Burstedt M, Sandgren O, Holmgren G, Forsman-Semb K (1999). Bothnia dystrophy caused by mutations in the cellular retinaldehyde-binding protein gene (RLBP1) on chromosome 15q26. Investig Ophthalmol Vis Sci.

[21] Hamel C, Dessalces E, Meunier I (2014). Retinitis punctata albescens. Inherited chorioretinal dystrophies.

[22] Burstedt MS, Mönestam E (2010). Self-reported quality of life in patients with retinitis pigmentosa and maculopathy of Bothnia type. Clin Ophthalmol (Auckland, NZ)..

[23] Lasch KE, Marquis P, Vigneux M, Abetz L, Arnould B, Bayliss M (2010). PRO development: rigorous qualitative research as the crucial foundation. Qual Life Res.

[24] US Department of Health and Human Services. Food and Drug Administration. 2009. Guidance for Industry: Patient-reported outcome measures: use in medical product development to support labeling claims. http://www.da.gov/downloads/drugs/guidanceComplianceRegulatoryInformation/Guidances/UCM193282.pdf. Accessed December 5, 2012.

[25] Mangione CM, Lee PP, Gutierrez PR, Spritzer K, Berry S, Hays RD (2001). Development of the 25-list-item national eye institute visual function questionnaire. Arch Ophthalmol.

[26] Mangione CM, Lee PP, Pitts J, Gutierrez P, Berry S, Hays RD (1998). Psychometric properties of the National Eye Institute visual function questionnaire (NEI-VFQ). Arch Ophthalmol.

[27] Owsley C, McGwin G, Scilley K, Kallies K (2006). Development of a questionnaire to assess vision problems under low luminance in age-related maculopathy. Investig Ophthalmol Vis Sci.

[28] Sloane M, Ball K, Owsley C, Bruni J, Roenker D (1992). The Visual Activities Questionnaire: developing an instrument for assessing problems in everyday visual tasks. Tech Dig Noninvasive Assess Vis Syst.

[29] Burstedt MS, Mönestam E, Sandgren O (2005). Associations between specific measures of vision and vision-related quality of life in patients with bothnia dystrophy, a defined type of retinitis pigmentosa. Retina..

[30] Mullins A, Burstedt M, Green J, Whelan J, Sloesen B, Ni X (2017). Cross-sectional evaluation of patient-reported outcomes (PROs) in patients with RLBP1 retinitis pigmentosa enrolled in a Natural History Study. Investig Ophthalmol Vis Sci.

[31] Francis JJ, Johnston M, Robertson C, Glidewell L, Entwistle V, Eccles MP (2010). What is an adequate sample size? Operationalising data saturation for theory-based interview studies. Psychol Health..

[32] Guest G, Bunce A, Johnson L (2006). How many interviews are enough? An experiment with data saturation and variability. Field Methods..

[33] Latham K, Baranian M, Timmis MA, Pardhan S (2015). Difficulties with goals of the Dutch ICF activity inventory: perceptions of those with retinitis pigmentosa and of those who support them. Investig Ophthalmol Vis Sci.

[34] Braun V, Clarke V (2006). Using thematic analysis in psychology. Qual Res Psychol.

[35] Hsieh HF, Shannon SE (2005). Three approaches to qualitative content analysis. Qual Health Res.

[36] Kerr C, Nixon A, Wild D (2010). Assessing and demonstrating data saturation in qualitative inquiry supporting patient-reported outcomes research. Expert Rev Pharmacoecon Outcomes Res.

[37] Patrick DL, Burke LB, Gwaltney CJ, Leidy NK, Martin ML, Molsen E (2011). Content validity—establishing and reporting the evidence in newly developed patient-reported outcomes (PRO) instruments for medical product evaluation: ISPOR PRO Good Research Practices Task Force report: part 2—assessing respondent understanding. Value Health..

[38] World Health Organisation. Change the definition of blindness. https://www.who.int/blindness/Change%20the%20Definition%20of%20Blindness.pdf. Accessed Dec 5, 2012.

[39] Sousa MC, Biteli LG, Dorairaj S, Maslin JS, Leite MT, Prata TS (2015). Suitability of the visual field index according to glaucoma severity. J Curr Glaucoma Pract.

[40] Hamel C (2006). Retinitis pigmentosa. Orphanet J Rare Dis.

[41] Rivolta C, Sharon D, DeAngelis MM, Dryja TP (2002). Retinitis pigmentosa and allied diseases: numerous diseases, genes, and inheritance patterns. Hum Mol Genet.

[42] Lowe J, Drasdo N (1992). Patients’ responses to retinitis pigmentosa. Optom Vis Sci.

[43] Senthil MP, Khadka J, Pesudovs K (2017). Seeing through their eyes: lived experiences of people with retinitis pigmentosa. Eye..

[44] Sugawara T, Hagiwara A, Hiramatsu A, Ogata K, Mitamura Y, Yamamoto S (2010). Relationship between peripheral visual field loss and vision-related quality of life in patients with retinitis pigmentosa. Eye..

[45] Finger RP, Fenwick E, Owsley C, Holz FG, Lamoureux EL (2011). Visual functioning and quality of life under low luminance: evaluation of the German Low Luminance Questionnaire. Investig Ophthalmol Vis Sci.

[46] Mills RP (1998). Correlation of quality of life with clinical symptoms and signs at the time of glaucoma diagnosis. Trans Am Ophthalmol Soc.

[47] Gothwal VK, Wright TA, Lamoureux EL, Pesudovs K (2009). Visual Activities Questionnaire: assessment of subscale validity for cataract surgery outcomes. J Cataract Refract Surg.

[48] Levinson RD, Monnet D, Yu F, Holland GN, Gutierrez P, Brezin AP (2009). Longitudinal cohort study of patients with birdshot chorioretinopathy. V. Quality of life at baseline. Am J Ophthalmol.

[49] Chung DC, McCague S, Yu ZF, Thill S, DiStefano-Pappas J, Bennett J, Cross D, Marshall K, Wellman J, High KA (2018). Novel mobility test to assess functional vision in patients with inherited retinal dystrophies. Clin Exp Ophthalmol.

[50] Russell S, Bennett J, Wellman JA, Chung DC, Yu Z-F, Tillman A (2017). Efficacy and safety of voretigene neparvovec (AAV2-hRPE65v2) in patients with RPE65-mediated inherited retinal dystrophy: a randomised, controlled, open-label, phase 3 trial. Lancet..

[51] Khadka J, McAlinden C, Pesudovs K (2013). Quality assessment of ophthalmic questionnaires: review and recommendations. Optom Vis Sci.

