# Supplementary material for: Qualitative Interviews to Better Understand the Patient Experience and Evaluate Patient-Reported Outcomes (PRO) in RLBP1 Retinitis Pigmentosa (RLBP1 RP)
Source: Adv Ther. 2020 May 5;37(6):2884–901. doi: 10.1007/s12325-020-01275-4 (PMC7467452; doi:10.1007/s12325-020-01275-4)
Supplement: Supplementary file 1 — Supplementary material 1 (DOCX 662 kb) [file 12325_2020_1275_MOESM1_ESM.docx]

**Qualitative interviews to better understand the patient experience and evaluate patient reported outcomes (PRO) in *RLBP1* retinitis pigmentosa (*RLBP1* RP)**

Jane Green^1^, Chloe Tolley^2^, Sarah Bentley^2^, Rob Arbuckle^2^, Marie Burstedt^3^, James Whelan^1^, Karen Holopigian^4^, Kali Stasi^5^, Brigitte Sloesen^6^, Claudio Spera^7^, Jean-Yves Deslandes^7^, Anmol Mullins^7^

^1^Memorial University of Newfoundland, St. John’s, Canada, ^2^Adelphi Values, Bollington, UK, ^3^University of Umeå, Sweden, ^4^Novartis Institute of Biomedical Research, East Hanover, NJ, USA, ^5^Novartis Institute of Biomedical Research, Cambridge, MA, USA, ^6^Novartis Pharmaceuticals Corporation, East Hanover, NJ, USA, ^7^Novartis Pharma AG, Basel, Switzerland

**Corresponding author:** Sarah Bentley, Adelphi Values Ltd, Adelphi Mill, Grimshaw Lane, Bollington, Cheshire, SK10 5JB. Tel: +44 1625 578686; Fax: +44 1625 577328; Email: [sarah.bentley@adelphivalues.com](mailto:sarah.bentley@adelphivalues.com)

#### Supplementary File 1: Detailed NEI VFQ-25 Cognitive Debriefing Results

**
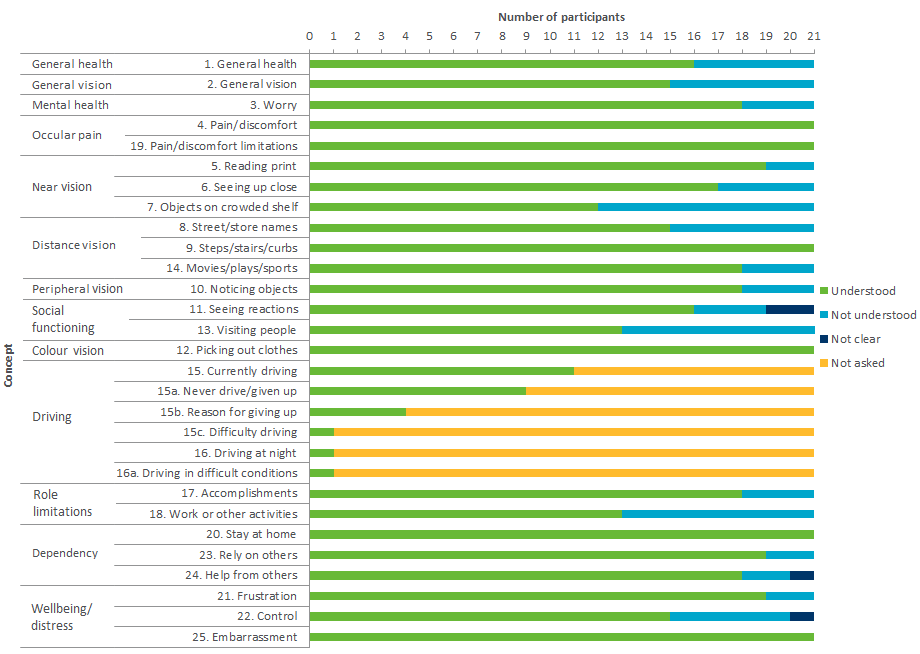
**

**Figure 1. Participant understanding of the NEI VFQ-25**


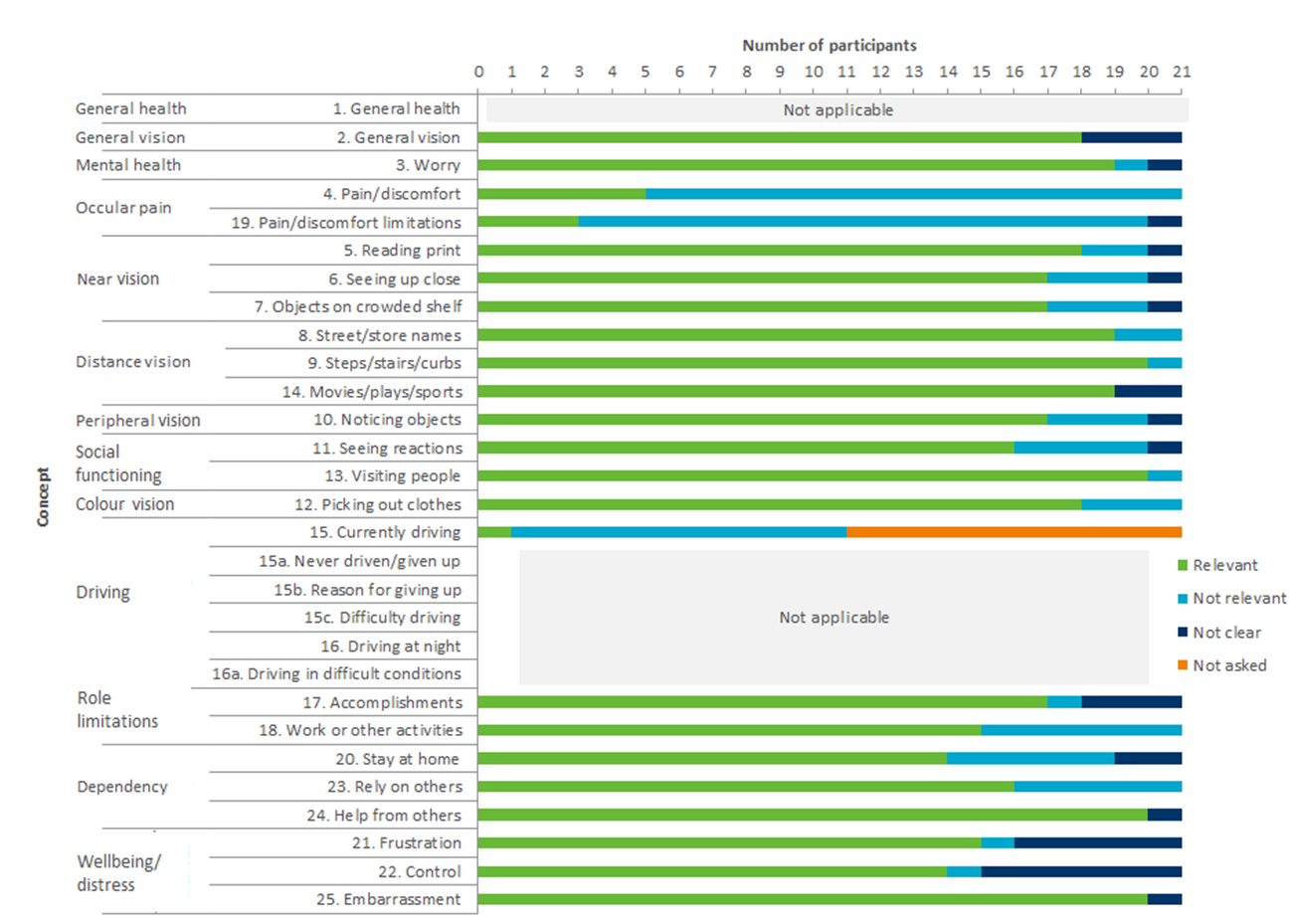


Figure 2. Relevance of concepts in the NEI VFQ-25

| Table 1. Detailed cognitive debriefing results for the NEI VFQ-25 | | | | |
| --- | --- | --- | --- | --- |
| Item | Understanding | Relevance | Response options | Comments |
| **Item 1**  **In general, would you say your overall health is:**  Excellent, very good, good, fair, poor | 16/21 understood the item without difficulty  5/21 had difficulty interpreting the item as they were unsure whether they should consider their eye condition when selecting a response: *“I don’t know if the eye illness should be taken into consideration”* Male, 20 | n/a | 14/21 understood the response options without difficulty  7/21 were not asked | 1/21 felt that this was a good question to start with: *“I think that's a good question to start with…Because it's very much an easy question, puts people at ease, and it gives you a baseline from which to look at your visual issues”* Male, 47 |
| **Item 2**  **At the present time, would you say your eyesight using both eyes, with glasses or contact lenses if you wear them, is:**  Excellent, good, fair, poor, or very poor, or are you completely blind? | 15/21 understood the item without difficulty  6/21 had difficulty knowing how to answer the item due to the number of different symptoms of *RLBP1* RP and being unable to compare their personal experiences to others: *“I don't know what the, what… because I don't know what they’re measuring it against, like that’s a difficult question to answer. Well like for me it's poor but maybe somebody else would consider it as good”* Female, 41, and because the lighting condition was not specified: “*Hard to know, how well can I see with both eyes, with this light? This is not a good question”* Male, 11 | 18/21 found the item to be relevant  3/21 did not clearly comment on relevance | 13/21 understood the response options without difficulty  3/21 found it difficult to select a response option as they struggled to compare their vision to ‘normal’ vision: “*In this case they need to assess – how much does a person with normal sight see? And then to assess, where am I compared to this person with normal vision”* Male, 54  1/21 did not clearly comment on the response options  4/21 were not asked | 1/21 felt that asking about being ‘completely blind’ was too abrupt: “*How funny at the end, or are you completely blind. So abrupt*” Female, 28  1/21 suggested that a numerical rating scale may be more appropriate: “*you perhaps need to grade the answers a bit better. Perhaps 1-10 or something like that. I don’t know, it’s very hard*” Male, 45 |
| **Item 3**  **How much of the time do you worry about your eyesight?**  None of the time, a little of the time, some of the time, most of the time, all of the time | 18/21 understood the item without difficulty  3/21 did not interpret the item as intended, did not worry about their condition or did not understand the timeframe: *“Depending on when in your life – where in life you are…Do they mean now – some of the time”* Male, 54 | 19/21 found the item to be relevant  1/21 did not find the item to be relevant as he did not worry: *“You might think about it, but it’s not worth it to worry”* Male, 45  1/21 did not clearly comment on relevance | 11/21 understood the response options without difficulty  3/21 did not clearly comment on the response options  7/21 were not asked | 1/21 felt that ‘worry’ was not the correct term to be used: *“I don’t worry because that’s like it is for me basically. Worry would be appropriate in terms of worrying about the future, how it will be in the future, get worse”* Male, 67 |
| **Item 4**  **How much pain or discomfort have you had in and around your eyes for example burning, itching or aching?**  None, mild, moderate, severe or very severe | 21/21 understood the item without difficulty | Only 5/21 found the item to be relevant, and even for those 5, they suggested the relevance was generally due to eye strain due to the use of contact lenses, this therefore indicates that eye pain is not a proximal symptom of *RLBP1* RP. All participants reporting relevance were from Canada.  16/21 did not find the item to be relevant since they did not experience pain or discomfort as part of their condition: *“I don’t know what it’s like to have pain in the eyes. If you get something in your eye I know what that’s like, but I don’t have this”* Male, 20 | 11/21 understood the response options without difficulty. Mild was described as *“Sometimes itching, no pain”* (F-65-Ca-VA(vs)-VF(vs)) and “*Just discomfort, no pain”* (M-50-Ca-VA(vs)-VF(vs)). Moderate was described as “*often itchy and dry, mostly itchy and dry*” Female, 41. Severe pain/discomfort was not experienced by any participants but was described as: “*like it’s not to the point where I'm always digging my, you know, always rubbing them or anything, but people will notice that I do rub my eyes a lot*” Female, 41  1/21 did not clearly comment on the response options  9/21 were not asked | 3/21 commented that discomfort was more relevant than pain, but that the discomfort was due to watching TV (M-47-Ca-VA(s)-VF(vs)), tired eyes (F-65-Ca-VA(vs)-VF(vs)) and from straining the eyes in the sun (F-35-Ca-VA(mo)-VF(vs)): *“Sometimes I've had itching and watering eyes. Uh, if I watch too much TV in the night*” Male, 47  1/21 reported that headaches were more relevant than pain or discomfort: *“Headaches. Um, I don’t get a lot of them, but when I get them they’re pretty severe and I know they’re just sort of visual-related*” Male, 40 |
| **Item 5**  **How much difficulty do you have reading ordinary print in newspapers?**  No difficulty at all, a little difficulty, moderate difficulty, extreme difficulty, stopped doing this because of your eyesight, stopped doing this for other reasons or not interested in doing this | 19/21 understood the item without difficulty  2/21 had difficulty interpreting the item as 1/21 felt that the item should specify if a reading aid is used: *“If you want to be updated on the aids, you might not read the traditional way. Unless it’s possible to magnify, which I do via the computer*” Male, 45 and 1/21 felt that the example could be broadened to ‘any print’: “*I can’t read any print, so I guess it could have expanded a little bit more there on that. They could have said “any print”, you know, ordinary or enhanced”* Female, 63 | 18/21 found the item to be relevant  2/21 did not find the item to be relevant as they were able to read in good lighting: *“For me that question is not relevant since I’m not visually impaired. I can read things if I have light”* Male, 20  1/21 did not clearly comment on relevance | 15/21 understood the response options without difficulty  2/21 did not clearly comment on the response options  4/21 were not asked | 2/21 felt that the response options that state ‘stopped doing this…’ should be removed since patients will not stop reading unless they are completely blind due to available reading aids: *“You can stop reading books, and there are alternatives. You can listen to books. So you can’t just say that you stop doing that”* Female, 52 |
| **Item 6**  **How much difficulty do you have doing work or hobbies that require you to see well up close, such as cooking, sewing, fixing things around the house, or using hand tools?**  No difficulty at all, a little difficulty, moderate difficulty, extreme difficulty, stopped doing this because of your eyesight, stopped doing this for other reasons or not interested in doing this | 17/21 understood the item without difficulty  4/21 had difficulty knowing how to respond to the item as the level of difficulty they experienced varied across the examples: *“It, it almost should be made into more than one question…Well, because some you can do and some you can’t”* Female, 65 | 17/21 found the item to be relevant  3/21 did not find the item to be relevant: *“You might think about it, but it’s not worth it to worry”* Male, 45  1/21 did not clearly comment on relevance | 14/21 understood the response options without difficulty  5/21 found it difficult to select a response option as they would never ‘stop’ doing the activities (3/5) or could select a different response option for the different examples provided (2/5)  2/21 were not asked | 3/21 suggested that the examples provided were outdated: *“The examples sounded a bit like college words from the 60s… Paying the bills. Everything is digital so there’s a lot of touch screens, and you can’t feel that*” Female, 58.  1/21 suggested that the ‘stopped doing this…’ options should be removed: *“You don’t stop doing things but you try to find other ways to do them”* Female, 52  2/21 reported that using hand tools and sewing were not relevant. Appropriate examples of tasks requiring the patient to see up close included cooking (n=3), using a computer (n=2), paying bills (n=2), fixing things around the house (n=1), reading/ writing (n=1) |
| **Item 7**  **Because of your eyesight, how much difficulty do you have finding something on a crowded shelf?**  No difficulty at all, a little difficulty, moderate difficulty, extreme difficulty, stopped doing this because of your eyesight, stopped doing this for other reasons or not interested in doing this | 12/21 understood the item without difficulty  9/21 had difficulty responding to the item as the lighting condition was not specified (3/9) and the familiarity of the scenario was not specified (6/9): “*I’d have to say moderate…You know, that's in my own home…That's not in someone else’s home and it’s not in the store…That's not out in the supermarket or a pharmacy. I wouldn't be able to find it. I’d have to get someone to help”* Female, 61 | 17/21 found the item to be relevant  3/21 did not find the item to be relevant as difficulty depended on the level of light or if they were in a shop rather than at home: *“If the question had mentioned light, if it’s difficult to find things on a crowded shelf when you come from outside. Or in a shop with poor lighting, is it difficult to find things, that would have been relevant”* Male, 25  1/21 did not clearly comment on relevance | 11/21 understood the response options without difficulty  1/21 had difficulty selecting a response option as she reported that the difficulty was in feeling the object rather than seeing the object: *“Because instead of not being able to see, you feel. So you don’t need to find things. So it’s a tough question and I don’t know how to answer”* Female, 61  8/21 were not asked  1/21 did not clearly comment on the response options | 1/21 felt that the ‘stopped doing this’ options should be removed but did not specify why |
| **Item 8**  **How much difficulty do you have reading street signs or the names of stores?**  No difficulty at all, a little difficulty, moderate difficulty, extreme difficulty, stopped doing this because of your eyesight, stopped doing this for other reasons or not interested in doing this | 15/21 understood the item without difficulty  6/21 reported a difference in reading street signs than reading store signs (see comments) | 19/21 found the item to be relevant  2/21 did not find the item to be relevant as they did not having difficulty reading signs: “*I’m relatively healthy so I don’t have these difficulties. It’s when it gets dark that it gets much harder*” Male, 20 | 8/21 understood the response options without difficulty  5/21 found it difficult to select a response option. 3/21 reported that it was difficult to select one answer as street signs and store signs were different, 1/21 had never been able to read street signs so found it difficult to select an answer and 1/21 reported that it depended on whether the sign was directly in front of her or off to the side: *“Now if I didn’t—if they were kind of—if the street signs were kind of off to the side I would have trouble because they’re not right in front of me”* Female, 32  7/21 were not asked  1/21 did not clearly comment on the response options | 3/21 found reading store signs easier than street signs: *“Street signs are almost impossible. I almost have to climb up to them. But shop signs are easier”* Male, 45  2/21 also commented that store signs are often lit up which can make them easier to read: *“99% of shop signs are lit up. Are they are in places with lighting. But street signs can be difficult”* Male, 25  1/21 commented that store signs can be different sizes and colors making them more difficult to read in some cases: “*Different colors, different, uh, background, but usually street signs are constant. I guess it's easier to pick -- pick that out because your eyes are used to seeing it”* Male, 29  1/21 commented that the item should specify if the sign is in front or off to the side Female, 32 |
| **Item 9**  **Because of your eyesight how much difficulty do you have going down steps, stairs, or curbs in dim light or at night?**  No difficulty at all, a little difficulty, moderate difficulty, extreme difficulty, stopped doing this because of your eyesight, stopped doing this for other reasons or not interested in doing this | 21/21 understood the item without difficulty | 20/21 found the item to be relevant  1/21 did not find the item to be relevant as she uses a cane and has learned to feel the ground when walking: “*You have your cane. If you have the condition you feel around, your legs, using cane, it’s not needed”* Female, 61 | 13/21 understood the response options without difficulty  1/21 had difficulty selecting a response option as they would require assistance going down steps/stairs/curbs: *“There has to be issues in any case but I need an assistant”* Female, 61  7/21 were not asked | 1/21 suggested that the item could be made more specific as stairs with bannisters were easier to use: *“The worst thing is a stair that has only a few steps. That lack the banister and so on. But if there’s a banister then there’s a stair it’s excellent”* Male, 67  2/21 felt that the ‘stopped doing this’ items should be removed as they would never be able to stop going down steps/stairs or curbs: *“You can’t stop using stairs because you can’t see”* Male, 25 |
| **Item 10**  **Because of your eyesight, how much difficulty do you have noticing objects off to the side while you are walking along?**  No difficulty at all, a little difficulty, moderate difficulty, extreme difficulty, stopped doing this because of your eyesight, stopped doing this for other reasons or not interested in doing this | 18/21 understood the item without difficulty  3/21 had difficulty interpreting the item as the lighting condition was not specified (2/3) or the wording was not clear Male, 67: *“If it’s light I can see them, if it’s dark, I can’t. So the question remains the same: is it light or dark?"* Male, 11 | 17/21 found the item to be relevant  3/21 did not find the item to be relevant as 2/21 did not experience too much difficulty with their peripheral vision and 1/21 was less concerned with their peripheral vision compared to other symptoms: *“Depending on how big the objects are, would I notice objects? I don’t think I’d have too much difficulty”* Male, 20  1/21 did not clearly comment on relevance | 14/21 understood the response options without difficulty  1/21 had difficulty selecting a response option as the ‘stopped doing this’ options were not relevant: *“This question is also a bit difficult to find answer options for”* Male, 45  3/21 were not asked  3/21 did not clearly comment on the response options | 6/21 reported that the ‘stopped doing this’ response options should be removed as they would never stop trying to notice objects off to the side: *“I’s a bit strange in relation to this question, but you understand what to answer”* Female, 28  2/21 suggested that an example of the item off to the side should be provided: *“can you find a yellow mailbox when you go to post a letter*…*do you bump into objects when you are out walking, do you see cycles that are sticking out, do you see a risk when you are out walking. Or connect it to an activity. Then the question is relevant”* Female, 58 |
| **Item 11**  **Because of your eyesight, how much difficulty do you have seeing how people react to the things you say?**  No difficulty at all, a little difficulty, moderate difficulty, extreme difficulty, stopped doing this because of your eyesight, stopped doing this for other reasons or not interested in doing this | 16/21 understood the item without difficulty  3/21 had difficulty interpreting the item as they were unsure if they should consider *“physical reaction of how they use their facial expressions…or if was just…their bodily reaction”* Male, 29, did know how to answer given the lighting condition is not specified, felt that seeing people in a group would be more difficult or were unsure whether the item was relating to facial or physical reactions  2/21 did not clearly comment on their understanding | 16/21 found the item to be relevant  4/21 did not find the item to be relevant as they could see other people’s reactions: “None in my case…It would be sad to stop doing that because of your sight” Male, 20  1/21 did not clearly comment on relevance | 12/21 understood the response options without difficulty  3/21 had difficulty selecting a response option as they felt that the difference between ‘moderate’ and ‘extreme’ was large: “*The same here with moderate and extremely – it’s a very big leap”* Male, 25  6/21 were not asked | 2/21 reported that the ‘stopped doing this’ response options should be removed as they would never stop trying to see the reaction of others: “*Even if I can’t see someone’s reaction I get a feeling instead. It works. But it’s very individual…You can never do that. You can never stop*” Male, 54 |
| **Item 12**  **Because of your eyesight how much difficulty do you have picking out and matching your own clothes?**  No difficulty at all, a little difficulty, moderate difficulty, extreme difficulty, stopped doing this because of your eyesight, stopped doing this for other reasons or not interested in doing this | 21/21 understood the item without difficulty | 18/21 found the item to be relevant  3/21 did not find the item to be relevant as they did not have difficulty matching their clothes: *“I have no difficulty in doing that”* Female, 35 | 16/21 understood the response options without difficulty  5/21 were not asked | 2/21 reported that the ‘stopped doing this’ response options should be removed as they would never stop picking out clothes: “*You can’t stop, you have to be part of society, it is so important”* Female, 61  1/21 suggested that patients may interpret the item differently with regards to considering color or pattern: *“This is my personal opinion, but it feels as though – I can more easily see patterns than colors. For me it’s less of a worry – but I would firstly put color when it comes to matching*” Female, 28  1/21 reported that some patients may use color detecting aids which may make this activity less challenging: “*There are also – there is a device that you press and it tells you the color*” Male, 45 |
| **Item 13**  **Because of your eyesight, how much difficulty you have visiting with people in their homes, at parties or in restaurants?**  No difficulty at all, a little difficulty, moderate difficulty, extreme difficulty, stopped doing this because of your eyesight, stopped doing this for other reasons or not interested in doing this | 13/21 understood the item without difficulty  9/21 had difficulty interpreting the item as 8/21 reported that the examples including in the item were different in that some were more challenging than others due to the level of familiarity: *“Restaurants I find bad…But in homes, friends, like I said, they know I’m coming, they’ve got an extra few lights on or things like that, and a strange home, yes, I’d, I’d have a lot of trouble…If it’s in my friends’ house, a moderate difficulty, but someone else’s would be extreme difficulty”* Male, 50, and 2/21 felt that the lighting condition should be specified: *“In very dark areas, dark pubs it’s worse…Parties at home can be, are often quite well-lit, since it’s in someone’s home. I could answer in two ways…if it’s bright everywhere I have no difficulty, but when it’s dark I have a lot of difficulty”* Male, 20 | 20/21 found the item to be relevant  1/21 did not find the item to be relevant as they had difficulties with eating rather than being in a restaurant: *“The restaurant is not an issue. It becomes an issue when you need to eat it”* Male, 67 | 14/21 understood the response options without difficulty  7/21 were not asked | 2/21 reported that the ‘stopped doing this’ response options should be removed as they would not stop socializing because of their condition: “*I have not stopped doing it due to my eyesight, or for other reasons. 6 is quite a destructive answer”* Male, 25 |
| **Item 14**  **Because of your eyesight how much difficulty do you have going out to see movies, plays or sports events?**  No difficulty at all, a little difficulty, moderate difficulty, extreme difficulty, stopped doing this because of your eyesight, stopped doing this for other reasons or not interested in doing this | 18/21 understood the item without difficulty  3/21 had difficulty interpreting the item and reported that the examples were too broad as they differed in the level of difficulty: *“Cinema is very good, theatre too. They are the same thing. Sports events are – I don’t know, I’ve never been to a sports event where I’ve had issues that I can remember. It’s a lit up environment”* Male, 25 | 19/21 found the item to be relevant  2/21 did not clearly comment on relevance | 13/21 understood the response options without difficulty  1/21 had difficulty selecting a response option as she could answer the question based on whether she was alone or with someone else: *“Because it’s just really two answers. If I’m with someone, it’s no big, no big deal, but if you’re by yourself there is”* Female, 65  6/21 were not asked  1/21 did not clearly comment on the response options | 1/21 reported not attending sports events and rarely watching plays: *“I don’t like sports. I go to the movies and I’ve been to two Christmas concerts”* (F-58-Sw-VA(vs)-VF(vs))  1/21 suggested that the item should specify if she should answer based on being with someone or alone: *“Because it’s just really two answers. If I’m with someone, it’s no big, no big deal, but if you’re by yourself there is*” Female, 65 |
| **Item 15**  **Are you currently driving at least once in a while?**  Yes, No | 11/21 understood the item without difficulty  10/21 were not asked (as they had previously indicated they did not drive) | 1/21 was currently driving Female, 32  10/21 did not currently drive: *“I didn’t drive, never did”* Female, 41  10/21 were not asked (as they had previously indicated that they did not drive) | 9/21 understood the response options without difficulty  12/21 were not asked | No comments |
| **Item 15a**  **Have you never driven a car or have you given up driving?**  Never drove, gave up | Of the 10/21 that were not currently driving, 9/10 understood the item without difficulty  1/10 was not asked | Of the 10/21 that were not currently driving, 5/10 had given up driving and 4/10 had never driven  1/10 was not asked | Of the 10/21 that were not currently driving, 9/10 understood the response options without difficulty  1/10 were not asked | No comments |
| **Item 15b**  **IF YOU GAVE UP DRIVING: Was that mainly because of your eyesight, mainly for some other reason, or because of both your eyesight and other reasons?**  Mainly eyesight, mainly other reasons, both eyesight and other reasons | Of the 5/21 who gave up driving, 4/5 understood the item without difficulty  1/5 was not asked | Of the 5/21 who gave up driving, 4/5 gave up driving because of their eyesight  1/4 was not asked | Of the 5/21 who gave up driving, 4/5 understood the response options without difficulty  1/4 was not asked | No comments |
| **Item 15c**  **IF CURRENTLY DRIVING: How much difficulty do you have driving during the daytime in familiar places? Would you say**  **you have:**  No difficulty at all, a little difficulty, moderate difficulty, extreme difficulty | The 1/21 currently driving understood the item without difficulty | The 1/21 currently driving found the item to be relevant | The 1/21 currently driving understood the response options without difficulty | No comments |
| **Item 16**  **How much difficulty do you have driving at night? Would you say you have:**  No difficulty at all, a little difficulty, moderate difficulty, extreme difficulty, stopped doing this because of your eyesight, stopped doing this for other reasons or not interested in doing this | The 1/21 currently driving understood the item without difficulty | The 1/21 currently driving found the item to be relevant | The 1/21 currently driving understood the response options without difficulty | No comments |
| **Item 16a**  **How much difficulty do you have driving in difficult conditions such as in bad weather, during rush hour, or on the freeway or in city traffic?**  No difficulty at all, a little difficulty, moderate difficulty, extreme difficulty, stopped doing this because of your eyesight, stopped doing this for other reasons or not interested in doing this | The 1/21 currently driving understood the item without difficulty | The 1/21 currently driving found the item to be relevant | The 1/21 currently driving had difficulty selecting a response options as she could have selected a different response for each of the examples listed:  *“Um, in weather-related, if it’s snowing oddly enough I’m good if it’s after dark because the snow illuminates everything. So my best time driving after dark is when the snow is down. Uh, because the pavement is black, snow is white. And if it’s raining I, I can’t drive at all. Um, but when its congested traffic, you know, I manage. It’s moderately—I’m just constantly having to check. So it’s not the same for all of them”* Female, 32 | Responses could vary for each of the examples listed (see response options) |
| **Item 17**  **Do you accomplish less than you would like to because of your vision?**  All of the time, most of the time, some of the time, a little of the time, or none of the time | 18/21 understood the item without difficulty  3/21 did not understand the item as 2/21 felt that ‘accomplish less’ was too broad and were unsure what tasks might fall into this category and 1/21 did not understand the term ‘accomplish’ (note that this was the 11 year old child participant): “*Like give an example or yeah. Like, I don’t know, do I accomplish little?...Is it in your daily life? Is it in your professional?”* Female, 41 | 17/21 found the item to be relevant  1/21 did not find the item to be relevant as she felt determined to not let her condition hold her back: *“Determination will get you anywhere”* Female, 41  3/21 did not clearly comment on relevance | 11/21 understood the response options without difficulty  3/21 found it difficult to select a response option as 2/21 felt that measuring accomplishment in regards to the amount of time was not appropriate and 1/21 felt that tasks can take longer with *RLBP1* RP so found it difficult to select an answer: *“What do they mean by all the time? The answers are a bit hard to define. I would redefine them”* Male, 25  7/21 were not asked | 2/21 commented that requiring more time to complete tasks was perhaps more important than accomplishing tasks: *“It’s just, you know, I would like to be able to go on home and clean the house in a normal timeframe or go get groceries on my own and be in and out in an hour”* Female, 32  1/21 felt that the item was too negatively worded: *“I’m sorry, but it doesn’t. It’s a horrible question”* Female, 41  1/21 reported that patients may blame their accomplishments on their visual condition and therefore the item may not collect accurate data: “*The problem is that it’s very easy to blame something else or the vision if you don’t do something you’d like to. I don’t know what to answer on this one…t doesn’t seem like a good question for a questionnaire”* Male, 20 |
| **Item 18**  **Are you limited in how long you can work or do other activities because of your vision?**  All of the time, most of the time, some of the time, a little of the time, or none of the time | 13/21 understood the item without difficulty  8/21 did not understand the item as 5/21 felt that the term ‘activities’ was too broad and required clarification, 2/21 participants responded to the item in terms of work only and 1/21 did not understand what ‘other activities’ meant (note that this was the 11 year old child participant): *“I think many people would answer the questions in different ways, depending on what you chose to incorporate”* Female, 28 | 15/21 found the item to be relevant  6/21 did not find the item to be relevant as they were not limited in the amount of time that they could carry out work or activities: *“I might get tired at the end of a workday because I’ve strained myself a lot. I don’t know if ‘how long’ is a very relevant question*” Female, 28 | 10/21 understood the response options without difficulty  2/21 found it difficult to select a response option as 1/21 could not pick an option as they were not working and 1/21 found it difficult to indicate the level of impact in terms of time: *“I don't like the rate, the scale that they're using there. Because some of the time, or most of the time – I guess I can only do some things for a certain amount of time”* Female, 41  6/21 were not asked  3/21 did not clearly comment on the response options | 3/21 reported that they may be limited but not necessarily in how long they could work/carry out activities: *“I think it’s a difficult question. My fitness level or energy level is not lowered. I function as before, but it takes longer to complete”* Female, 58  1/21 reported that she had adapted her job which had reduced the impact: *“So—but right now I’m not limited because my job allows me to do dayshifts and then I’m home”* Female, 32  1/21 also commented that work was different to other activities: *“Yeah, I think work is different than—because work doesn't generally take the same amount of strain on my eyes as, as watching TV”* Male, 47 |
| **Item 19**  **How much does pain or discomfort in or around your eyes, for example burning, itching, or aching keep you from doing what you’d like to be doing?**  All of the time, most of the time, some of the time, a little of the time, or none of the time | 21/21 understood the item without difficulty | 3/21 found the item to be relevant (2/21 described that this was related to eye strain when staring at a screen)  17/21 did not find the item to be relevant as they did not experience pain or discomfort as part of *RLBP1* RP: *“None of the time, because like I said, it’s not really even—burning and itching I haven’t had”* Female, 32  1/21 did not clearly comment on relevance | 10/21 understood the response options without difficulty  10/21 were not asked  1/21 did not clearly comment on the response options | No comments |
| **Item 20**  **I stay at home most of the time because of my eyesight**  Definitely true, mostly true, not sure, mostly false, definitely false | 21/21 understood the item without difficulty | 14/21 found the item to be relevant  5/21 did not find the item to be relevant as they did not stay at home because of their visual condition: *“That question is no fun. I don’t think people stay at home because of their eyesight”* Female, 52. However, 2/21 reported that the item may be relevant for other patients.  2/21 did not clearly comment on relevance | 16/21 understood the response options without difficulty  3/21 were not asked  2/21 did not clearly comment on the response options | 2/21 commented that the item does not assess the challenges associated with going out: *“I think it gets the point across, but, you know, again I think what it doesn’t do is, is capture the ordeal that going places often is”* Male, 40  1/21 commented that the item is not clear on whether staying inside means inside the home or within the land of the property: “*So, if it's staying at my own property, if I go out of my own driveway, uh, I'm -- I'm -- you know, I'm good with that. But staying -- staying at home inside my own house, not going outside, that's a different -- that's a different answe*r” Male, 65 |
| **Item 21**  **I feel frustrated a lot of the time because of my eyesight**  Definitely true, mostly true, not sure, mostly false, definitely false | 19/21 understood the item without difficulty  2/21 did not understand the item as they were unsure how to answer (see ‘response options’) | 15/21 found the item to be relevant  1/21 did not find the item to be relevant as she did not feel frustrated because of her condition Female, 41  5/21 did not clearly comment on relevance | 13/21 understood the response options without difficulty  2/21 had difficulty selecting a response option as  they felt that true/false were not the most appropriate descriptors to describe the level of frustration experienced: *“I don’t like the true or false…I would go like some of the time, most of the time, all, you know what I'm saying”* Female, 41  6/21 were not asked | 1/21 commented that the item should state ‘most of the time’ rather than ‘a lot of the time’ to be consistent with the previous item: *“There’s no difference, it would be better to have the same so that the answers answer the same level”* Female, 28 |
| **Item 22**  **I have much less control over what I do because of my eyesight**  Definitely true, mostly true, not sure, mostly false, definitely false | 15/21 understood the item without difficulty  5/21 had difficulty interpreting the item as 3/21 reported that the item was too broad and the specific activities which require control should be stated and 2/21 did not understand what ‘less control over what I do’ meant: *“Much less control seems unclear. Control over “what” you do should be asked instead. This might be a good question, but it needs to be specified control over just what it is exactly”* Male, 11  1/21 did not clearly comment on their understanding | 14/21 found the item to be relevant  1/21 did not find the item to be relevant as he had adapted to living with the condition: “*But if you live with this all the time – you use other ways, you see with your hands”* Male, 45  6/21 did not clearly comment on relevance | 9/21 understood the response options without difficulty  2/21 did not understand the response options as 1/21 did not understand the item and therefore how to answer the item (F-41-Ca-VA(s)-VF(vs) ) and 1/21 did not understood the ‘mostly true’ response option: *“True or false. I don’t know what partially true is”* Male, 20  10/21 were not asked | 1/21 thought that a five-point scale would be easier to respond to: *“I need to answer on a scale from 1 to 5”* Male, 20 |
| **Item 23**  **Because of my eyesight, I have to rely too much on what other people tell me**  Definitely true, mostly true, not sure, mostly false, definitely false | 19/21 understood the item without difficulty  2/21 found it difficult to interpret the item as 1/21 felt that it was unclear: *“It could be a good question but it should be clearer”* (M-11-Sw-VA(mi)-VF(mi)) and 1/21 reported that he was more reliant on other people at night than in the day: *“In the daytime I don’t, but, uh, in the night time I probably would, yeah”* Male, 47 | 16/21 found the item to be relevant  5/21 did not find the item to be relevant as they did not have to rely on others. 2/21 also mentioned that the item may be more appropriate in cases where patients are completely blind: *“Because – if you were to go completely blind, then it would be critical*” Male, 54 | 8/21 understood the response options without difficulty  11/21 were not asked  2/21 did not clearly comment on the response options | No comments |
| **Item 24**  **I need a lot of help from others because of my eyesight**  Definitely true, mostly true, not sure, mostly false, definitely false | 18/21 understood the item without difficulty  2/21 found it difficult to interpret the item as 1/21 reported that she was more reliant on other people at night than in the day (F-32-Ca-VA(mo)-VF(mo)) and 1/21 reported that she required more help from others when outside of their home: *“Mostly outside the home…I get help inside the home but I can do a lot of things that I don't need help with”* Female, 65  1/21 did not clearly comment on their understanding | 20/21 found the item to be relevant  1/21 did not clearly comment on relevance | 8/21 understood the response options without difficulty  11/21 were not asked  2/21 did not clearly comment on the response options | 1/21 reported that item 23 and 24 were addressing the same concept: *“This one is much better, you could remove 23 and replace it with 24*” Male, 11 |
| **Item 25**  **I worry about doing things that will embarrass myself or others because of my eyesight**  Definitely true, mostly true, not sure, mostly false, definitely false | 21/21 understood the item without difficulty | 20/21 found the item to be relevant  1/21 did not clearly comment on relevance | 8/21 understood the response options without difficulty  11/21 were not asked  2/21 did not clearly comment on the response options | 1/21 thought that response options from the previous section which focus on the amount of time would be easier to respond to: *“I still think it should be “all of the time, most of the time, some of the time, rarely”* Female, 41  1/21 commented that embarrassing others may not be as relevant: “*But I don’t know if I would include embarrassing others, I don’t quite know what it means”* Female, 28 |
