# Supplementary material for: Qualitative Interviews to Better Understand the Patient Experience and Evaluate Patient-Reported Outcomes (PRO) in RLBP1 Retinitis Pigmentosa (RLBP1 RP)
Source: Adv Ther. 2020 May 5;37(6):2884–901. doi: 10.1007/s12325-020-01275-4 (PMC7467452; doi:10.1007/s12325-020-01275-4)
Supplement: Supplementary file 2 — Supplementary material 2 (DOCX 508 kb) [file 12325_2020_1275_MOESM2_ESM.docx]

**Qualitative interviews to better understand the patient experience and evaluate patient reported outcomes (PRO) in *RLBP1* retinitis pigmentosa (*RLBP1* RP)**

Jane Green^1^, Chloe Tolley^2^, Sarah Bentley^2^, Rob Arbuckle^2^, Marie Burstedt^3^, James Whelan^1^, Karen Holopigian^4^, Kali Stasi^5^, Brigitte Sloesen^6^, Claudio Spera^7^, Jean-Yves Deslandes^7^, Anmol Mullins^7^

^1^Memorial University of Newfoundland, St. John’s, Canada, ^2^Adelphi Values, Bollington, UK, ^3^University of Umeå, Sweden, ^4^Novartis Institute of Biomedical Research, East Hanover, NJ, USA, ^5^Novartis Institute of Biomedical Research, Cambridge, MA, USA, ^6^Novartis Pharmaceuticals Corporation, East Hanover, NJ, USA, ^7^Novartis Pharma AG, Basel, Switzerland

**Corresponding author:** Sarah Bentley, Adelphi Values Ltd, Adelphi Mill, Grimshaw Lane, Bollington, Cheshire, SK10 5JB. Tel: +44 1625 578686; Fax: +44 1625 577328; Email: [sarah.bentley@adelphivalues.com](mailto:sarah.bentley@adelphivalues.com)

#### Supplementary File 2: Detailed LLQ Cognitive Debriefing Results


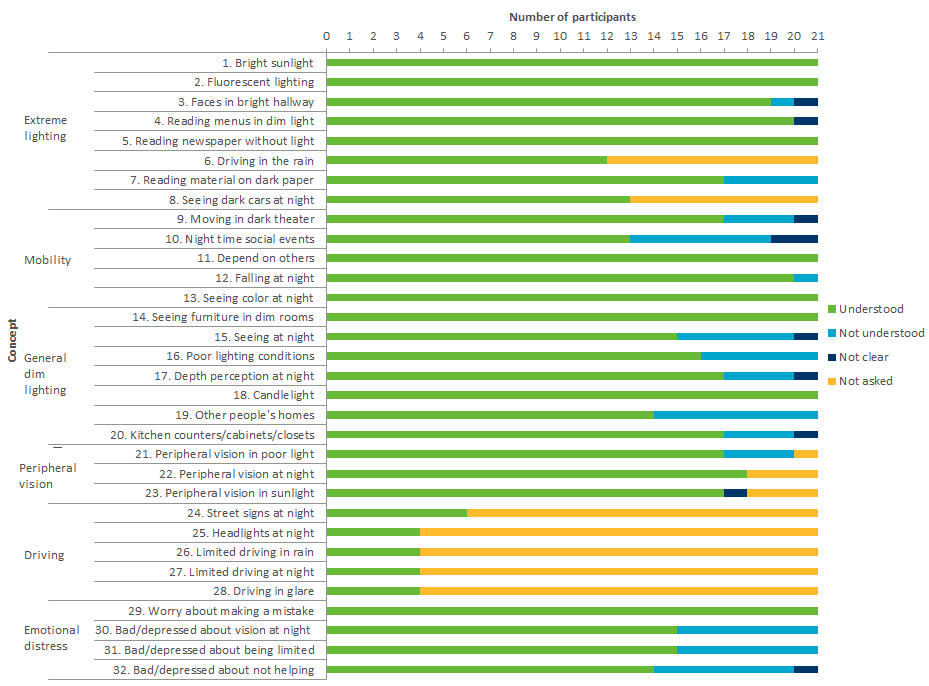


Figure 1. Participant understanding of the LLQ


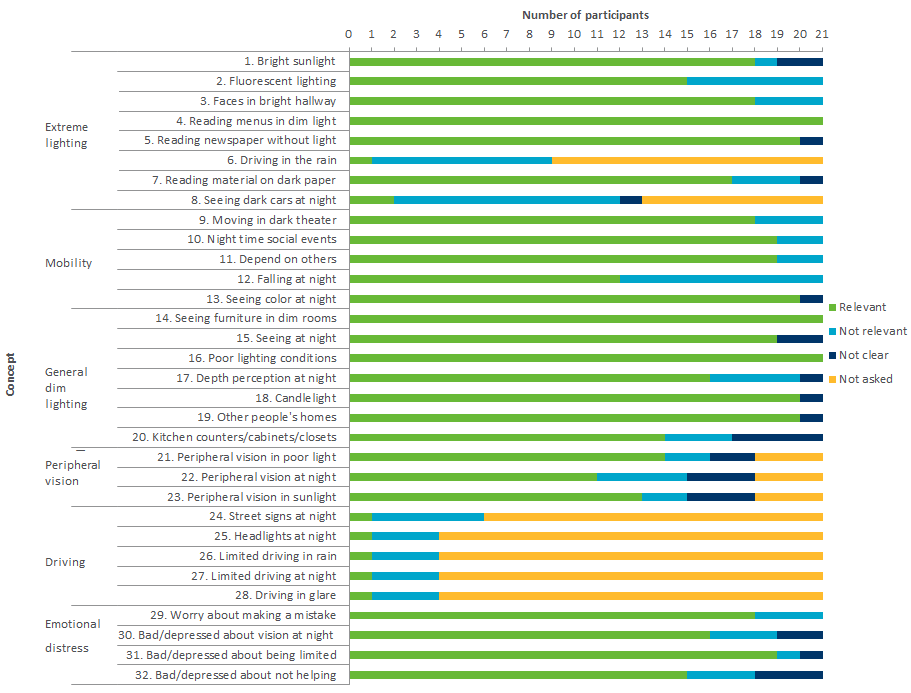


**Figure 2. Relevance of concepts in the LLQ**

| Table 1. Detailed LLQ cognitive debriefing results | | | | |
| --- | --- | --- | --- | --- |
| Item | Understanding | Relevance | Response options | Comments |
| **Item 1**  **Do you have difficulty seeing in bright sunlight?**  No difficulty at all, a little difficulty, some difficulty, a lot of difficulty, completely blind under these conditions, stopped going out in bright sunlight because of your vision, stopped for other reasons | 21/21 understood the item without difficulty | 18/21 found the item to be relevant  1/21 did not find the item to be relevant as the concept was not experienced by the individual: *“I don’t know if people, if others have this difficulty”* Male, 25  2/21 did not clearly comment on relevance | 14/21 understood the response options without difficulty  4/21 were not asked  3/21 did not clearly comment on the response options | 4/22 felt that the ‘stopped going out’ response option should be removed: *“That’s not what you do, you don’t stop…Even if you’re blind you still go outdoors”* Male, 54  2/22 felt that there should be an option for those who are completely blind: *“Because if it’s completely a completely blind person, this, uh, don’t fit”* Female, 58  1/22 reported that the context should be clarified: *“If you’re walking around during the day, I have no difficulty with that. But if I’ve been indoors in the dark and then suddenly. Sunlight hurts the eyes”* Male, 20 |
| **Item 2**  **Do you have difficulty seeing in fluorescent lighting, like that found in stores and offices?**  No difficulty at all, a little difficulty, some difficulty, a lot of difficulty, completely blind under these conditions, stopped going to places with fluorescent lighting because of your vision, stopped for other reasons | 21/21 understood the item without difficulty | 15/21 found the item to be relevant  6/21 did not find the item to be relevant as they did not experience difficulties in fluorescent lighting: *“Fluorescent light? Like those we have in school. Maybe others do have problems seeing, I don’t”* Male, 11 | 13/21 understood the response options without difficulty  8/21 were not asked | 4/21 commented that the ‘stopped’ response options should be removed as patients would be unlikely to stop going to places with fluorescent lighting: *“I think the answer, ‘have stopped doing this’ is strange…you don’t stop doing things, you find other ways or solutions or it takes longer, you compensate, or use aids*” Female, 28 |
| **Item 3**  **Do you have difficulty seeing people’s faces in a hallway when direct sunlight is behind them?**  No difficulty at all, a little difficulty, some difficulty, a lot of difficulty, completely blind under these conditions, stopped doing this because of your vision, stopped for other reasons | 19/21 understood the item without difficulty  1/21 had difficulty interpreting the item as he said he had to turn his head away from the bright light: *“Well, if I get sunlight in my eyes, I can’t just, but I can look this way - then you can see. That question, maybe a clearer definition would be better”* Male, 11. Note that this was the 11-year-old child participant.  1/21 did not clearly comment on understanding | 18/21 found the item to be relevant  3/21 did not find the item to be relevant as 1/21 would turn his head away in direct sunlight (M-11-Sw-VA(mi)-VF(mi)), 1/21 had not had this experience (M-50-Sw-VA(s)-VF(s)) and 1/21 would not be able to see a person’s face in any light: “*It doesn’t matter, I have difficulty seeing no matter what. I see only the contours of someone”* Male, 67 | 10/21 understood the response options without difficulty  11/21 were not asked | 2/21 commented that the ‘stopped’ response options should be removed as patients would be unlikely to stop trying to see in bright lighting: *“I don’t like - have stopped doing this due to my vision…I think it should be enough with I’m completely blind under these conditions. It’s fairly concrete*” Male, 20  1/21 suggested that the item may not be specific to visual impairment since all people have difficulty seeing a bright sunlight: “*Well, everyone has difficulty seeing in that type of light…if you have strong light in front of you, you see the light”* Male, 25 |
| **Item 4**  **Do you have difficulty reading menus in dimly lit restaurants?**  No difficulty at all, a little difficulty, some difficulty, a lot of difficulty, completely blind under these conditions, stopped doing this because of your vision, stopped for other reasons, never reads menus | 20/21 understood the item without difficulty  1/21 did not clearly comment on understanding | 21/21 found the item to be relevant | 11/21 understood the response options without difficulty  2/21 had difficulty interpreting the response options as they were unsure how to answer between ‘completely blind’ and ‘stopped because of vision’ since they meant the same: *“Completely blind under these conditions then, because I can’t read any menus…I can’t do it. So yeah, I guess stopped, isn’t it?...Well, I don’t know, yeah, somebody reads it to me”* Female, 41  8/21 were not asked | 4/21 reported that ‘completely blind’ and ‘stopped because of vision’ response options had the same meaning: *“You know, sometimes I have two answers, I guess”* Female, 63  1/21 felt that the stopped for other reasons was not appropriate: *“Also here, answer 7, have stopped doing this for other reasons. What other reasons. If you can’t see then you can’t see, that’s the only reason. Although, if you’re severely dyslexic and have this that might be for other reasons”* Male, 25  1/21 felt that the ‘never reads menus’ option was not appropriate: *“Answer 8 says don’t read menus. That’s also strange. Of course you try to read a menu, if you have an aid. Or some sort of help”* Female, 52 |
| **Item 5**  **Do you have difficulty reading the newspaper without good lighting?**  No difficulty at all, a little difficulty, some difficulty, a lot of difficulty, completely blind under these conditions, stopped doing this because of your vision, stopped for other reasons, never read the newspaper | 21/21 understood the item without difficulty | 20/21 found the item to be relevant  1/21 did not clearly comment on the relevance of the item | 9/21 understood the response options without difficulty  11/21 were not asked  1/21 did not clearly comment on understanding of the response options | 2/21 felt that items 4 and 5 were asking the same thing: *“Similar to the one before with the menu”* Female, 63  2/21 commented that the ‘stopped’ and ‘never reads’ response options should be removed as patients would be unlikely to stop trying to read: *“The answers are good up to number 4”* Female, 52  1/21 commented that knowing what was meant by ‘good lighting’ was difficult when she had never had good vision: *“When you haven't got very much vision, it’s hard to say what’s good lighting”* Female, 65 |
| **Item 6**  **Do you get upset because you have difficulty seeing while driving in the rain at night?**  Don’t have difficulty with rain at night, get upset none of the time, a little of the time, some of the time, most or all of the time, stopped doing because of your vision, stopped for other reasons, never drove | 9/21 understood the item without difficulty  12/21 were not asked (as they had previously indicated that they did not drive) | 1/21 found the item to be relevant Female, 32  8/21 did not find the item to be relevant as they did not drive: *“That one does not -- that does not apply to me. I don't drive in the rain. I don’t drive at all”* Female, 65  12/21 were not asked (as they had previously indicated that they did not drive) | 4/21 understood the response options without difficulty  17/21 were not asked | 1/21 commented that vision in rainy conditions is better than brighter conditions: *“The, the conditions that come with rain, sort of the dullness and stuff that’s actually a little better for me”* Male, 40 |
| **Item 7**  **Do you have difficulty reading material printed on dark colored paper?**  No difficulty at all, a little difficulty, some difficulty, a lot of difficulty, completely blind under these conditions, stopped doing this because of your vision, stopped for other reasons, never read material printed on dark colored paper | 17/21 understood the item without difficulty  4/21 had difficulty interpreting the item and commented that difficulty being able to read would be dependent upon the level on contrast: “*Um, if the wording is, uh, a bright -- closer to white or gray, I don't find any difficulty…But if it's colored, then I find it will be*” Male, 29 | 17/21 found the item to be relevant  3/21 did not find the item to be relevant as they did not tend to read material on dark colored paper: *“It’s not relevant as long as we’re not talking about studies. Since – print on dark colored paper, it doesn’t feel as though that occurs”* Male, 20  1/21 did not clearly comment on relevance | 7/21 understood the response options without difficulty  14/21 were not asked | - 2/21 commented that the ‘stopped’ and ‘never reads’ response options should be removed as patients would be unlikely to stop trying to read: “*It works, but it’s almost the same as – have stopped doing that, is the same thing as never read…There are two answers that mean the same thing”* Female, 28 |
| **Item 8**  **Do you have difficulty seeing dark colored cars while driving at night?**  No difficulty at all, a little difficulty, some difficulty, a lot of difficulty, completely blind under these conditions, stopped doing this because of your vision, stopped for other reasons, never drove | 13/21 understood the item without difficulty  8/21 were not asked (as they had previously indicated that they did not drive) | 2/21 found the item to be relevant (1/21 talked about past experiences of driving)  10/21 did not find the item to be relevant as they did not drive: *“That one does not -- that does not apply to me. I don't drive in the rain. I don’t drive at all”* Female, 65  8/21 were not asked (as they had previously indicated that they did not drive)  1/21 did not clearly comment on relevance | 5/21 understood the response options without difficulty  16/21 were not asked | - 1/21 commented that the item was not appropriate given that cars have headlights which make them visible: *“This is strange since cars have lights, and if you see them you know there’s a car so you don’t walk out in front of it”* Male, 11 |
| **Item 9**  **Because of your vision, are you bothered that you have difficulty moving around in a darkened theater?**  Don’t have difficulty getting around, bothered none of the time, a little of the time, some of the time, most or all of the time, stopped doing this because of your vision, stopped for other reasons | 17/21 understood the item without difficulty  3/21 had difficulty interpreting the item as they were thinking about the difficulty of the task rather than how much it bothered them when answering the item: *“I thought it was just asking me, uh, how - how it affects me - in the night. So - I guess I - I overlooked that the bother - the bother part of it”* Male, 29  1/21 did not clearly comment on understanding | 18/21 found the item to be relevant  3/21 did not find the item to be relevant as 1/21 had never been to the theater (M-50-Sw-VA(s)-VF(s)), 1/21 had more difficulty finding his seat than moving around (M-67-Sw-VA(vs)-VF(vs)), and 1/21 was not bothered about having difficulty moving around: *“It’s asking if you, you’re in a theatre and it’s dark, does it bother you that you can’t get around? It doesn't bother me”* Female, 65 | 10/21 understood the response options without difficulty  1/21 did not understand how to answer using the response options as  he had difficulty with moving around but it did not bother him: *“That's an interesting question, because I do have difficulty most of the time moving around in a dark theater, but I'm not bothered by it”* Male, 47  10/21 were not asked | 1/21 reported that she experienced difficulty finding her seat as more so than moving around in a theater: *“So the issue is not moving, but finding the row and where to sit in the first place is”* Male, 67  1/21 commented that ‘darkened theatre’ may not be modern but that it was still understood: *“Darkened theatre, is that what it’s called? Perhaps it is. The word sounded unmodern, but you understand that it’s if you are in some sort of performance where you’re sitting in a cinema or dance performance”* Female, 28 |
| **Item 10**  **Because of your vision, do you have difficulty going out to nighttime social events such as sporting events, the theater, friend’s homes, church, or restaurants?**  No difficulty at all, a little difficulty, some difficulty, a lot of difficulty, completely blind under these conditions, stopped doing this because of your vision, stopped for other reasons | 13/21 understood the item without difficulty  6/21 had difficulty interpreting the item as they felt that the item was too broad since the examples used differed in the level of difficulty: *“A sports event wouldn't be much difficulty, whereas the theater would be more or a restaurant would be more…Yeah, so, again, you might want to consider breaking that down”* Male, 47. 4/21 also reported that the examples used in the item can vary in the level of light, which would have implications for the response option selected: “*They’re very varied when to comes to light. Sporting events are quite bright, while theatres and churches can be a bit darker*” Male, 20  2/21 did not clearly comment on understanding | 19/21 found the item to be relevant  2/21 did not clearly comment on relevance | 10/21 understood the response options without difficulty  11/21 were not asked | 1/21 suggested that familiar locations should be separated from unfamiliar locations: *“I think it’s important to separate familiar environments and unfamiliar environments. In a familiar environment I can make my way, I can use the mobility service transportation, go to the entrance and meet my friends. If I’ve never been there before it’s more difficult”* Female, 58  1/21 suggested that the examples should be updated: *“It also feels old. It’s a broad span of examples, but they might not be the most common things…Meet up with friends, sports events work. But church, of course people do go, but it’s not a very modern example of a social event”* Female, 28  1/21 commented that ‘friends’ should be removed as an example as they help to make the visit easier: *“I wouldn't add friends to that question because any friend know what you like anyway so they’re going to do, make it possible and appropriate for you to come there anyway”* Male, 50 |
| **Item 11**  **Do you depend on others to help you because of your vision at night or under poor lighting?**  None of the time, a little of the time, some of the time, most or all of the time, stopped going out under these conditions because vision causes you to  be dependent on others, stopped for other reasons | 21/21 understood the item without difficulty | 19/21 found the item to be relevant  2/21 did not clearly comment on relevance | 10/21 understood the response options without difficulty  1/21 had difficulty interpreting the response options as she did not know how to answer when requiring help all of the time: “*Yes, but I don’t like the answers. It says never. It should maybe say always…When I’m out in the evening I always need to have someone with me”* Female, 52  10/21 were not asked | 1/21 commented that many patients need help during the day as well as at night: *“A lot of people with this disease need help from others also during the day*” Female, 28  1/21 felt that depending on others should be changed to ‘requiring an assistant’: “*But to get to where you’re going, then you need an assistant. So really you would need to rephrase the question to if you need an assistant or not”* Male, 67 |
| **Item 12**  **Do you worry or are you concerned that you might fall at night because of your vision?**  None of the time, a little of the time, some of the time, most or all of the time, stopped doing because vision causes you to worry about falling at night, stopped for other reasons | 20/21 understood the item without difficulty  1/21 had difficulty interpreting the item as she was unsure if she should answer thinking about being alone or with someone else: *“I’m thinking about when I go out at night, I’m always with someone…They should say if you’re alone – if they mean alone, they should say alone”* Female, 65 | 12/21 found the item to be relevant  9/21 did not find the item to be relevant as they did not worry about falling: *“That's not saying that I might not fall, it's just that I'm not worried about it”* Male, 47 | 10/21 understood the response options without difficulty  11/21 were not asked | 1/21 reported that falling over may not just be related to vision but also to age: *“But then you can ask many elderly why they don’t dare going out at night, they are like what?”* Male, 67 |
| **Item 13**  **Do you have difficulty seeing colors at night?**  No difficulty at all, a little difficulty, some difficulty, a lot of difficulty, completely blind under these conditions, stopped doing this because of your vision, stopped for other reasons | 21/21 understood the item without difficulty | 20/21 found the item to be relevant  1/21 did not clearly comment on relevance | 8/21 understood the response options without difficulty  1/21 had difficulty interpreting the response options as she did not see at all during the night time and so could not comment on color vision: *“So I don‘t see anything at night anyway so I don’ t know what to answer”* Male, 20  10/21 were not asked | 7/21 commented that color vision was affected in day light as well as at night time: *“The colors are of course not as clear. Yes, it’s a relevant question. Although seeing colors can also be affected during the day”* Female, 28  4/21 reported that the ‘stopped’ response options should be removed as they would not stop trying to see color: “*It’s not an active choice to stop doing it. It’s not an activity you stop doing – either you can see or you cannot”* Female, 28 |
| **Item 14**  **Do you have difficulty seeing furniture in dimly lit rooms with dark floors?**  No difficulty at all, a little difficulty, some difficulty, a lot of difficulty, completely blind under these conditions, stopped doing this because of your vision, stopped for other reasons | 21/21 understood the item without difficulty | 21/21 found the item to be relevant | 12/21 understood the response options without difficulty  9/21 were not asked | 2/21 reported that the ‘stopped’ response options should be removed as they would not stop trying to see furniture: “*The one, have stopped doing this for other reasons is – have you stopped seeing furniture in a dimly lit room with dark floors – I don’t know what other reason there could be”* Male, 25  2/21 reported that the furniture color can make a difference in the ability to detect the contrast: *“That’s also about contrasts. It depends on what color the furniture is. If it’s a dark floor and a grey or white leather sofa I see it, the contours”* Female, 58 |
| **Item 15**  **Do you have difficulty seeing at night?**  No difficulty at all, a little difficulty, some difficulty, a lot of difficulty, completely blind under these conditions | 15/21 understood the item without difficulty  5/21 had difficulty interpreting the item and commented that the item should be more specific to state whether they should answer based on being indoors or outdoors: “*When it’s pitch black outside, or are you indoors at night and seeing?”* Male, 54  1/21 did not clearly comment on understanding | 19/21 found the item to be relevant  2/21 did not clearly comment on relevance | 8/21 understood the response options without difficulty  13/21 were not asked | No comments |
| **Item 16**  **Do you have difficulty seeing in poor lighting conditions such as at dusk or dawn or in a poorly lit room?**  No difficulty at all, a little difficulty, some difficulty, a lot of difficulty, completely blind under these conditions | 16/21 understood the item without difficulty  5/21 had difficulty interpreting the items and reported that there were differences in their visual ability in dusk, dawn and poorly lit rooms: *“Well, dawn’s – dusk is harder than dawn because the daylight is breaking up, so dusk is a little bit harder than dawn”* Male, 50 | 21/21 found the item to be relevant | 8/21 understood the response options without difficulty  13/21 were not asked | No comments |
| **Item 17**  **Do you have difficulty with depth perception at night?**  No difficulty at all, a little difficulty, some difficulty, a lot of difficulty, completely blind under these conditions | 17/21 understood the item without difficulty  3/21 did not understand the item as they did not understand the term ‘depth perception’: *“What is depth perception? I’ve never heard that word”* Female, 52  1/21 did not clearly comment on understanding | 16/21 found the item to be relevant  4/21 did not find the item to be relevant as they did not experience problems with depth perception: *“Depth perception isn’t something that has affected me”* Male, 20  1/21 did not clearly comment on relevance | 7/21 understood the response options without difficulty  14/21 were not asked | 3/21 reported that they experience difficulties with depth perception in the day and at night, whereas 2/21 specified that they cannot judge depth at night due to poor night vision: *“Um, I can, you know, if there’s sunlight I’ve got no trouble with depth but if there’s no light I can’t see”* Female, 65  3/21 suggested that the term ‘depth perception’ could be rephrased: “*Yes, I would rather write see at a distance or estimate distance”* Female, 28  1/21 commented that “*instead of completely blind it should perhaps say, don’t have depth perception*” Male, 25 |
| **Item 18**  **Do you have difficulty seeing in candlelight?**  No difficulty at all, a little difficulty, some difficulty, a lot of difficulty, completely blind under these conditions, stopped going to places with candlelight  because of your vision, stopped for other reasons | 21/21 understood the item without difficulty | 20/21 found the item to be relevant  1/21 did not clearly comment on relevance | 10/21 understood the response options without difficulty  1/21 had difficulty selecting a response option as her vision improves over time: “*I’m blind in the beginning but when I’m there for a while I can see, I can see okay*” Female, 65  1/21 did not clearly comment on the response options  9/21 were not asked | - 2/21 suggested that the ‘stopped’ response options should be removed: *“6 and 7 should be removed”* Male, 20 |
| **Item 19**  **Do you have difficulty seeing when you visit other people’s homes because there is not enough light?**  No difficulty at all, a little difficulty, some difficulty, a lot of difficulty, completely blind under these conditions, stopped doing this because of your vision, stopped for other reasons | 14/21 understood the item without difficulty  7/21 had difficulty interpreting the item stating that their visual ability is dependent on how familiar the environment is: “*But where I go most people know and the lights are on, so, you know, I would say a lot of difficulty based on if I were to go somewhere and it was dimly light, somebody else’s house, unfamiliar surroundings it would be a lot of, a lot of difficulty”* Female, 32 | 20/21 found the item to be relevant  1/21 did not clearly comment on relevance | 8/21 understood the response options without difficulty  1/21 did not clearly comment on the response options  12/21 were not asked | 1/21 commented that the lighting outside of the home can impact her visual ability inside of the home: *“if I’m coming, going in someone’s house in the daylight with sunshine, it’s very difficult, but if it’s in the night-time with dim lights it’s not as difficult because I’m out in the dim light”* Female, 65  1/21 suggested that the ‘stopped’ response options should be removed: *“6 and 7 should be removed”* Male, 20 |
| **Item 20**  **Do you have difficulty seeing under kitchen counters or in cabinets or closets because there is not enough light?**  No difficulty at all, a little difficulty, some difficulty, a lot of difficulty, completely blind under these conditions, stopped doing this because of your vision, stopped for other reasons | 17/21 understood the item without difficulty  3/21 had difficulty interpreting the item as the level of difficulty would vary depending on if the environment was familiar or not: *“Like if you’re talking about your own house or someone else’s house or, like I said, at my place I’ve got lights in all my closet*” Male, 50  1/21 did not clearly comment on understanding | 14/21 found the item to be relevant  3/21 did not find the item to be relevant because lights can be used in cabinets and closets: *“I have lights in my closets. That I turn on…I can’t answer that question, it doesn’t apply to me”* Female, 41  4/21 did not clearly comment on relevance | 4/21 understood the response options without difficulty  2/21 had difficulty selecting a response option as they did not know how to answer when their cabinets/closets have lights in them: *“I can’t answer that question, it doesn’t apply to me”* Female, 41  15/21 were not asked | - 4/21 stated that additional lighting may be used: *“That’s the same for everyone but I have a flashlight”* Male, 20 |
| **Item 21**  **Do you have difficulty with your peripheral vision under poor lighting conditions?**  No difficulty at all, a little difficulty, some difficulty, a lot of difficulty, completely blind under these conditions | 17/21 understood the item without difficulty  3/21 did not understand the item as 2/21 did not understand the term ‘peripheral vision’ and 1/21 did not understand why peripheral vision was being assessed when central vision was affected: *“Do you see less to the sides when you have RP? I can see as well straight ahead as well as to the sides”* Male, 11  1/21 was not asked | 14/21 found the item to be relevant  2/21 did not find the item to be relevant as 2/21 did not experience problems with peripheral vision: *“If you have RP with peripheral loss, that’s where this question is more – I don’t have that”* Male, 45  2/21 did not clearly comment on relevance  3/21 were not asked | 2/21 understood the response options without difficulty  2/21 were not asked | 3/21 commented that the term ‘peripheral vision’ should be reworded: *“But most easy would be to include see to the sides*” Female, 61  1/21 commented that the difficulties with peripheral vision exist in all lighting conditions: “*A lot, but, but in, in all conditions*” Male, 40  1/21 suggested that poor light could have different meanings: “*For me it could be bright light, if I see poorly in that type of light. It depends what you incorporate into it*” Female, 28 |
| **Item 22**  **Do you have difficulty with your peripheral vision at night?**  No difficulty at all, a little difficulty, some difficulty, a lot of difficulty, completely blind under these conditions | 18/21 understood the item without difficulty  3/21 were not asked | 11/21 found the item to be relevant  4/21 did not find the item to be relevant as 3/21 did not experience problems at night time at 1/21 was completely blind at night time: *“Completely blind under these conditions, don't see at night”* Female, 65  3/21 did not clearly comment on relevance  3/21 were not asked | 5/21 understood the response options without difficulty  16/21 were not asked | 3/21 felt that item 22 and item 21 were the same: *“Poor lighting conditions and at night are the same. That’s how I interpret it*” Male, 54  1/21 commented that the difficulties with peripheral vision exist in all lighting conditions Male, 40  1/21 felt that ‘at night’ was more easily interpreted than ‘poor lighting conditions’: *“It depends what you incorporate into it. But ‘at night’ is easier to interpret”* Female, 28 |
| **Item 23**  **Do you have difficulty with your peripheral vision in bright sunlight?**  No difficulty at all, a little difficulty, some difficulty, a lot of difficulty, completely blind under these conditions | 17/21 understood the item without difficulty  1/21 did not clearly comment on understanding  3/21 were not asked | 13/21 found the item to be relevant  2/21 did not find the item to be relevant as they did not experience problems with peripheral vision: “*To me it’s irrelevant but I don’t know if others have difficulty”* Male, 25  3/21 did not clearly comment on relevance  3/21 were not asked | 7/21 understood the response options without difficulty  14/21 were not asked | 1/21 felt that the three items relating to peripheral vision should be reduced to one question: “*In that case it should be one question. To ask if you have this issue overall”* Female, 52 |
| **Item 24**  **Do you have difficulty reading street signs when driving at night?**  No difficulty at all, a little difficulty, some difficulty, a lot of difficulty, completely blind under these conditions, stopped doing this because of your vision, stopped for other reasons, never drove | 6/21 understood the item without difficulty  15/21 were not asked as they had previously indicated that they did not drive | 1/21 found the item to be relevant (F-32-Ca-VA(mo)-VF(mo))  5/21 did not find the item to be relevant as they did not drive: *“I don’t drive, so it’s not applicable*” Female, 41  15/21 were not asked as they had previously indicated that they did not drive | 3/21 understood the response options without difficulty  18/21 were not asked as they had previously indicated that they did not drive | No comments |
| **Item 25**  **While driving at night, do headlights from oncoming cars cause you difficulty?**  No difficulty at all, a little difficulty, some difficulty, a lot of difficulty, completely blind under these conditions, stopped doing this because of your vision, stopped for other reasons, never drove | 4/21 understood the item without difficulty  17/21 were not asked as they had previously indicated that they did not drive | 1/21 found the item to be relevant Female, 32  5/21 did not find the item to be relevant as they did not drive: “*Don't, don't drive at night”* Male, 50  15/21 were not asked as they had previously indicated that they did not drive | 3/21 understood the response options without difficulty  18/21 were not asked as they had previously indicated that they did not drive | No comments |
| **Item 26**  **Have you limited driving in the rain at night because of difficulty seeing?**  No difficulty at all, a little difficulty, some difficulty, a lot of difficulty, completely blind under these conditions, stopped doing this because of your vision, stopped for other reasons, never drove | 4/21 understood the item without difficulty  17/21 were not asked as they had previously indicated that they did not drive | 1/21 found the item to be relevant Female, 32  5/21 did not find the item to be relevant as they did not drive: *“No, same, don't drive”* Male, 50  15/21 were not asked as they had previously indicated that they did not drive | 1/21 understood the response options without difficulty  20/21 were not asked as they had previously indicated that they did not drive | No comments |
| **Item 27**  **Do you limit your driving at night due to your vision?**  No difficulty at all, a little difficulty, some difficulty, a lot of difficulty, stopped doing this because of your vision, stopped for other reasons, never drove | 4/21 understood the item without difficulty  17/21 were not asked as they had previously indicated that they did not drive | 1/21 found the item to be relevant  5/21 did not find the item to be relevant as they did not drive: *“No, same, don't drive”* Male, 50  15/21 were not asked as they had previously indicated that they did not drive: “*Never driven*” Female, 65 | 1/21 understood the response options without difficulty  20/21 were not asked as they had previously indicated that they did not drive | No comments |
| **Item 28**  **Do you have difficulty seeing while driving at dawn or dusk because of glare?**  No difficulty at all, a little difficulty, some difficulty, a lot of difficulty, completely blind under these conditions, stopped doing this because of your vision, stopped for other reasons, never drove | 4/21 understood the item without difficulty  17/21 were not asked as they had previously indicated that they did not drive | 1/21 found the item to be relevant  5/21 did not find the item to be relevant as they did not drive: *“No, same, don't drive”* Male, 50  15/21 were not asked as they had previously indicated that they did not drive: “*Never driven*” Female, 65 | 1/21 understood the response options without difficulty  20/21 were not asked as they had previously indicated that they did not drive | No comments |
| **Item 29**  **Do you worry or are you concerned that you may make a mistake at a social event because you can’t see well enough under poor lighting conditions? (for example, getting food on a fork, recognizing people, or reading the menu in a dimly lit restaurant)**  None of the time, a little of the time, some of the time, most or all of the time, stopped going to social events because concerned about making  a mistake, stopped for other reasons | 21/21 understood the item without difficulty | 18/21 found the item to be relevant  3/21 did not find the item to be relevant as they were not concerned about making a mistake: *“It’s been a while now, so I just don’t care anymore”* Female, 63 | 8/21 understood the response options without difficulty  2/21 did not clearly comment on the response options  11/21 were not asked | 1/21 reported that the ‘stopped’ response options should be removed as they would not stop going to events: “*All the answers that say have stopped doing it can be removed. It sounds as though – as though you’re sitting at home*” Female, 52 |
| **Item 30**  **Do you feel bad or depressed about your ability to see at night or under poor lighting conditions?**  None of the time, a little of the time, some of the time, most or all of the time, stopped going out under these conditions because feel bad or depressed about your ability to see, stopped for other reasons | 15/21 understood the item without difficulty  6/21 had difficulty interpreting the item since ‘bad’ and ‘depressed’ had different meanings, with feeling bad being milder than feeling depressed: “*Because bothered by something – you know, it bothers you. But being depressed about something is totally different things*” Female, 41 | 16/21 found the item to be relevant  3/21 did not find the item to be relevant as they did not feel bad or depressed: “*I don't really feel bad or depressed about it*” Male, 47  2/21 did not clearly comment on relevance | 6/21 understood the response options without difficulty  1/21 did not clearly comment on the response options  14/21 were not asked | 1/21 felt that ‘bad’ and ‘depressed’ were the same: *“No, I’d say they’re the same. Like I do, I gets depressed over a lot of it, believe me. I very easily get depressed over it”* Male, 50  2/21 suggested that the term ‘depressed’ should be reworded since it may be confused with milder feelings: “*Uh, most people probably think that depressed is just feeling down…so you might want to, you know, might consider rewording that”* Male, 47  2/21 suggested that ‘sad and frustrated’ may be more appropriate terms to use: *“I can feel that, I get frustrated and then I feel sad. Not depressed”* Female, 52 |
| **Item 31**  **Do you feel bad or depressed because your vision at night or under poor lighting keeps you from doing all that you would like to do?**  Not limited by vision at night or under poor lighting, feel bad none of the time, a little of the time, some of the time, most or all of the time, stopped trying to do things because feel bad or depressed about  your vision under these conditions, stopped for other reasons | 15/21 understood the item without difficulty  6/21 had difficulty interpreting the item since ‘bad’ and ‘depressed’ had different meanings | 19/21 found the item to be relevant  1/21 did not find the item to be relevant as she did not stop doing things because of poor vision at night: *“I don’t know, I think that you cannot stop living because you can’t see in the dark*” Female, 52  1/21 did not clearly comment on relevance | 9/21 understood the response options without difficulty  1/21 did not clearly comment on the response options  11/21 were not asked | 1/21 commented that the item should be more specific about what it is at night time that respondents should be thinking about: “*I understand the question, but it’s too general, I think. And it’s so difficult – what is it you’re wanting to do at night that you might get so upset about that you can’t do it”* Male, 45  1/21 commented that ‘depressed’ should be reworded: “*Uh, sometimes, sometimes, but, again, the depressed changed that”* Male, 47 |
| **Item 32**  **Do you feel bad or depressed that you aren’t able to help others as much as you want because of your vision at night or under poor lighting?**  None of the time, a little of the time, some of the time, most or all of the time, stopped trying to help others because feel bad or depressed  about your vision under these conditions, stopped for other reasons | 14/21 understood the item without difficulty  6/21 had difficulty interpreting the item since ‘bad’ and ‘depressed’ had different meanings  1/21 did not clearly comment on understanding | 15/21 found the item to be relevant  3/21 did not find the item to be relevant as 2/21 did not feel depressed and 1/21 felt that the item was irrelevant for those who cannot see: *“This one’ not needed at all, if you can’t see then what’s the point? You can’t help anyone else either*” Female, 61  3/21 did not clearly comment on relevance | 8/21 understood the response options without difficulty  1/21 did not understand the response options  12/21 were not asked | 1/21 reported that the ‘stopped’ response options should be removed as she would not want to stop trying to help others: “*If it was very dark outside and I saw someone on a bicycle fall over – I’d want to help them but wouldn’t be able to*” Male, 25 |
