# Supplementary material for: Qualitative Interviews to Better Understand the Patient Experience and Evaluate Patient-Reported Outcomes (PRO) in RLBP1 Retinitis Pigmentosa (RLBP1 RP)
Source: Adv Ther. 2020 May 5;37(6):2884–901. doi: 10.1007/s12325-020-01275-4 (PMC7467452; doi:10.1007/s12325-020-01275-4)
Supplement: Supplementary file 3 — Supplementary material 3 (DOCX 405 kb) [file 12325_2020_1275_MOESM3_ESM.docx]

**Qualitative interviews to better understand the patient experience and evaluate patient reported outcomes (PRO) in *RLBP1* retinitis pigmentosa (*RLBP1* RP)**

Jane Green^1^, Chloe Tolley^2^, Sarah Bentley^2^, Rob Arbuckle^2^, Marie Burstedt^3^, James Whelan^1^, Karen Holopigian^4^, Kali Stasi^5^, Brigitte Sloesen^6^, Claudio Spera^7^, Jean-Yves Deslandes^7^, Anmol Mullins^7^

^1^Memorial University of Newfoundland, St. John’s, Canada, ^2^Adelphi Values, Bollington, UK, ^3^University of Umeå, Sweden, ^4^Novartis Institute of Biomedical Research, East Hanover, NJ, USA, ^5^Novartis Institute of Biomedical Research, Cambridge, MA, USA, ^6^Novartis Pharmaceuticals Corporation, East Hanover, NJ, USA, ^7^Novartis Pharma AG, Basel, Switzerland

**Corresponding author:** Sarah Bentley, Adelphi Values Ltd, Adelphi Mill, Grimshaw Lane, Bollington, Cheshire, SK10 5JB. Tel: +44 1625 578686; Fax: +44 1625 577328; Email: [sarah.bentley@adelphivalues.com](mailto:sarah.bentley@adelphivalues.com)

#### Supplementary File 3: Detailed VAQ Cognitive Debriefing Results

**Figure 1. Participant understanding of the four VAQ items**

Figure 2. Relevance of the four VAQ items

| Table 1. Detailed VAQ cognitive debriefing results | | | | |
| --- | --- | --- | --- | --- |
| Item | Understanding | Relevance | Response options | Comments |
| **Item 1**  **I have problems adjusting to bright room lighting, after the room lighting has been rather dim**  Never, seldom, sometimes, often, never | 19/21 understood the item without difficulty  1/21 did not understand the item the first time that it was read: “*I had a hard time understanding it at first”* Female, 35  1/21 did not clearly comment on understanding | 17/21 found the item to be relevant  4/21 did not find the item to be relevant as difficulties adapting from bright to dark lighting were not experienced: *“That has never happened…it’s not relevant to me”* Male, 25 | 9/21 understood the response options without difficulty  12/21 were not asked | 1/21 suggested that the term ‘problems’ should be defined: *“Define problems. It still takes 10 seconds then I’ve sorted it out…You can be blinded, but is that really a problem if it’s for such a short period of time?”* Male, 20  1/21 suggested that the term ‘blinding’ would be more appropriate: *“Adjust your eyes you can’t ever do that...Blinding the word it might be”* Female, 61 |
| **Item 12**  **It takes me a long time to adjust to darkness after being in**  **bright light**  Never, rarely, sometimes, often, always | 21/21 understood the item without difficulty | 21/21 found the item to be relevant | 5/21 understood the response options without difficulty  15/21 were not asked  1/21 did not clearly comment on the response options | No comments |
| **Item 23**  **It takes me a long time to adjust to bright sunshine after I have been inside a building for a lengthy period of time**  Never, rarely, sometimes, often, always | 21/21 understood the item without difficulty | 17/21 found the item to be relevant  4/21 did not find the item to be relevant as they did not experience difficulty adjusting to bright sunshine, 2/21 commented that they did however have difficulty going from bright light to dim light: *“If I’m indoors I’m inside, but when I go outside, that’s when I have to adjust to a different light”* Female, 52 | 7/21 understood the response options without difficulty  14/21 were not asked | No comments |
| **Item 28**  **I have trouble adjusting from bright to dim lighting, such as**  **when going from daylight into a dark movie theater**  Never, rarely, sometimes, often, always | 21/21 understood the item without difficulty | 21/21 found the item to be relevant | 5/21 understood the response options without difficulty  16/21 were not asked | 1/21 commented that a more familiar example should be used since respondents may not have visited a movie theatre for a while: “*Yes, and you can exchange it for my own hall. When you see movie theater, you have to think back to the last time you went, what was it like. If you don’t go to the movies it doesn’t work. Take a better environment that you are in everyday*” Female, 58 |
